# Integrated CNN–LSTM–XGBoost hybrid model predicts shale oil seismic attributes and global oil price trends

**DOI:** 10.1038/s41598-026-55910-1

**Published:** 2026-06-04

**Authors:** Guoqing Chen, Tianwen Zhao, Cong Pang, Piyapatr Busababodhin

**Affiliations:** 1 Mathematical Modeling Research Center, Chengdu Jincheng College, Chengdu, 611731 China; 2 Department of Trade and Logistics, Daegu Catholic University, Gyeongsan, 38430 Republic of Korea; 3 Institute of Seismology, CEA, Wuhan, 40071 China; 4 Department of Mathematics, Faculty of Science, Mahasarakham University, Maha Sarakham, 44150 Thailand

**Keywords:** Oil price forecasting, Shale oil seismic attributes, Multi-modal deep learning, CNN-LSTM-XGBoost, Geoeconomic modeling, Energy science and technology, Mathematics and computing, Solid Earth sciences

## Abstract

This study proposes a hybrid CNN–LSTM–XGBoost model that integrates shale oil seismic attributes with macroeconomic indicators to predict global oil prices. The model extracts spatial features from seismic volumes using 3D CNNs and captures temporal dependencies in economic data via LSTM, with fused features processed by XGBoost regression. It achieves an RMSE of 3.61 USD/barrel, R^2^ of 0.847, and MAPE of 6.98%, outperforming standalone models by 12.7–23.1%. SHAP analysis shows that seismic attributes contribute an average marginal impact of 0.19 on the model output. The model uses seismic attributes as input features to forecast oil price trends, providing a transparent and interpretable framework for integrated geophysical–economic analysis. At the local scale, the model assists shale oil operators in optimizing drilling and production decisions by linking seismic-derived reservoir quality to medium-term supply expectations. At the broader global scale, the framework demonstrates how geological information can be systematically incorporated into energy market forecasting, offering a new paradigm for geoeconomic modeling under supply‑side uncertainties.

## Introduction

In light of profound transformations in the global energy landscape, shale oil resources have become a key strategic factor affecting energy security and market balance. In recent years, the North American shale revolution has significantly changed the global crude oil supply structure, making the correlation between shale oil production fluctuations and oil price trends increasingly stronger^1^. As a representative of unconventional oil and gas resources, shale oil development has the characteristics of short investment cycle and flexible production capacity adjustment, which makes its production changes have a rapid impact on short-term oil prices. However, it must be clarified that seismic attributes do not directly predict oil prices in real time; rather, they provide static-to-semi-static estimates of reservoir quality and potential productivity over monthly to quarterly scales^2^. These estimates influence medium-term supply expectations, which interact with OPEC + decisions and geopolitical events to affect global oil prices. The causal chain—seismic attributes → shale productivity potential → supply expectation adjustment → oil price movement—involves inevitable lags and noise, and our model is designed to capture statistical correlations within this cascade rather than instantaneous causality. At the same time, international oil price fluctuations, as an important indicator of the macro-economy, not only directly affect the fiscal revenue of oil-producing countries and the energy costs of consuming countries, but also trigger changes in inflation expectations through industrial chain transmission, which in turn has a profound impact on monetary policy formulation and energy strategy adjustment^3^. In this complex system, seismic exploration data, as the core basis for shale oil reservoir evaluation, contains rich geological structure information and fluid distribution characteristics^4^. Through multi-attribute fusion analysis, it can effectively reveal reservoir physical properties and production capacity potential, providing a unique perspective for understanding the transmission mechanism of "geological characteristics-production capacity changes-market response".

Existing research still has significant methodological limitations in the field of shale oil prediction and oil price modeling. Traditional statistical models and machine learning methods often have difficulty effectively capturing the multi-scale spatial features contained in seismic data, and lack the ability to model the nonlinear time series dynamics of oil prices^5–7^. When dealing with cross-domain correlations between "geology and economy", single model architectures generally face problems such as insufficient feature expression and weak spatiotemporal coupling modeling, resulting in insufficient stability of prediction results. Especially under extreme market conditions, traditional linear models have limited response capabilities to black swan events and cannot adapt to complex scenarios with drastic fluctuations in oil prices^8^. Most existing studies separate geological indicators from economic variables for analysis, fail to establish a unified feature interaction framework, ignore the marginal impact mechanism of shale oil production capacity changes on the supply and demand balance, and restrict the practical value of prediction models^9^.

This study proposes an innovative multimodal hybrid modeling framework that deeply integrates the advantages of three algorithms: convolutional neural network, long short-term memory network, and gradient boosting decision tree to build an end-to-end shale oil seismic attribute and oil price trend prediction system^10,11^. This framework realizes the collaborative encoding of spatial features of seismic images and macroeconomic time series for the first time, uses CNN to extract high-order representations of reservoir structures, captures the temporal dependencies of oil price fluctuations through LSTM, and finally establishes cross-domain associations with the help of XGBoost’s powerful feature fusion capabilities. Compared with existing methods, the main breakthroughs of this study are reflected in three aspects: first, a joint embedding method for seismic attribute bodies and market variables is developed to solve the problem of spatiotemporal alignment of heterogeneous data; second, a dynamic feature importance weighting mechanism is designed to enhance the model’s sensitivity to key driving factors; third, an uncertainty quantification module is introduced to provide reliability assessment for prediction results. This innovative architecture not only improves the accuracy and robustness of oil price forecasts, but also provides new analytical tools for understanding the complex interactive relationship between geological characteristics and energy markets.

## Overview of related work

In the development history of shale oil prediction methods, early research mainly relied on geostatistics and rock physics analysis methods. Traditional methods such as random forests and gradient boosting trees are widely used in reservoir parameter prediction, but these methods often have difficulty in dealing with the high-dimensional nonlinear characteristics of seismic data^12,13^. With the rise of deep learning technology, researchers began to explore the application of neural networks in shale oil prediction, especially the automatic feature extraction of seismic attributes. However, most of these methods are limited to static analysis at a single time point and fail to fully consider the dynamic change characteristics during shale oil development, resulting in insufficient continuity of prediction results in the time dimension.

Oil price prediction research has evolved from traditional econometric models to machine learning methods. Although early time series models such as ARIMA and GARCH can capture the basic fluctuation characteristics of oil prices, they perform poorly in dealing with nonlinear relationships and external shocks^14–16^. In recent years, machine learning methods such as support vector machines and neural networks have been introduced into the field of oil price prediction, significantly improving the model’s ability to adapt to complex market environments^17^. In particular, LSTM networks have shown unique advantages in capturing the long-term and short-term dependencies of oil prices, and can effectively model complex factors such as market sentiment transmission and policy shocks^18,19^. However, these methods generally ignore the intrinsic connection between energy markets and geological characteristics, resulting in limited predictive capabilities of the models in economic-geological coupling systems.

The application of convolutional neural networks in seismic attribute analysis has brought new technical paths to shale oil prediction^20^. Through its local connection and weight sharing characteristics, CNN can automatically learn spatial patterns in seismic data, such as faults, fractures, and reservoir boundaries. Key geological features. Studies have shown that deep CNN architectures can extract higher-order representations that are more discriminative than traditional manual features, significantly improving the accuracy of reservoir parameter prediction^21^. In particular, when processing three-dimensional seismic data, 3D CNN can better maintain the spatial structure information of the data, providing more reliable technical support for shale oil production capacity assessment^22^. However, these methods usually require a large amount of labeled data for supervised training, and face the challenge of high data acquisition costs in practical applications.

The XGBoost algorithm has been widely used in the field of energy prediction due to its excellent feature selection and nonlinear modeling capabilities^23^. The algorithm controls the complexity of the model through regularization terms, effectively preventing overfitting and performing well when processing high-dimensional features. In oil price forecasting, XGBoost can automatically identify key economic indicators and quantify their impact, providing an explainable decision basis for market analysis^24,25^. At the same time, its parallel computing characteristics enable the model to efficiently process large-scale data sets, which is particularly important for energy market forecasting with high real-time requirements. However, XGBoost has inherent limitations when processing spatiotemporal data, making it difficult to fully explore the spatiotemporal dependencies in the data^26^.

The latest progress in model integration strategies provides new ideas for the fusion analysis of multi-source heterogeneous data. Studies have shown that the complementary advantages of different algorithms can significantly improve prediction performance. In particular, the combination of deep learning and traditional machine learning can not only retain the spatiotemporal characteristics of data, but also enhance the generalization ability of the model. In the energy field, some studies have attempted to combine CNN with LSTM for production forecasting, or integrate LSTM with XGBoost for price analysis, but these methods often use a simple serial structure and fail to fully explore the interactive relationship between cross-modal features^27–32^. The latest research on ensemble learning has begun to focus on deep fusion at the feature level, and has achieved adaptive weighting of features from different data sources through attention mechanisms, which has pointed out the direction for building more powerful prediction systems^33–35^.

## Methodology

Considering the complex nonlinear spatiotemporal coupling relationship between shale oil seismic attributes and global oil price trends, we comprehensively collected multi-source heterogeneous data to build a prediction data system that integrates space, time and economy.

### Data and preprocessing

The data in this study are divided into three categories: shale oil seismic attribute data, global oil price historical data, and external auxiliary variables, as follows:

#### Seismic attribute data set

Two-dimensional and three-dimensional seismic exploration datasets from major shale oil blocks in North America, including the Bakken Basin and Permian Basin, were used in this study. The datasets cover key well site areas from 2010 to 2024, with a sampling interval of 2 ms and a spatial resolution of 25 × 25 m, and include multiple seismic attribute volumes such as RMS amplitude, frequency attenuation, and coherence. All datasets had undergone inversion processing prior to analysis. The Bakken Basin data were obtained from the North Dakota Industrial Commission (NDIC) GIS portal under a public-use license, comprising eight independent 3D seismic surveys covering 1,247 wells, while the Permian Basin data were sourced from the Texas Railroad Commission (RRC) in collaboration with the SMU Geothermal Laboratory under an academic non-commercial license, including twelve independent 3D seismic surveys covering 2,103 wells; both datasets were accessed in March 2024. To ensure consistency in model training and comparative analysis, all seismic volumes were standardized to a unified grid size of 512 × 512 × 64 voxels.

Table 1 summarizes the statistical characteristics of the seven key seismic attributes used in this study, reflecting the seismic data characteristics of major shale oil blocks in North America (Bakken Basin and Permian Basin) from 2010 to 2024. The sampling interval of this dataset is 2 ms, and the spatial dimensions are 512 × 512 × 64, which has a high temporal and spatial resolution, ensuring that the model can capture the coupling relationship between microstructural changes and macro reservoir evolution. From the perspective of mean and standard deviation, the amplitude RMS (mean 0.235, standard deviation 0.081) and amplitude envelope (mean 0.312, standard deviation 0.089) fluctuate strongly within the time window, indicating that there are obvious energy differences and attenuation characteristics inside the shale formation, which helps to reveal the development of fractures and reservoir heterogeneity; while the coherence coefficient (mean 0.745, standard deviation 0.092) is generally high, with a maximum value close to 0.997, indicating that the seismic reflection events inside the target block are continuous, providing a reliable basis for structural interpretation and fault identification.

**Table 1:** Summary of statistical characteristics of shale oil seismic attributes (2010–2024, major shale areas in North America).

Attribute name	Data type	Spatial dimensions (X × Y × Z)	Time coverage	Sampling interval	Average	Standard deviation	Maximum	Minimum	Unit
RMS amplitude	Continuous	512 × 512 × 64	2010–2024	2 ms	0.235	0.081	0.812	0.012	—
Dominant frequency	Continuous	512 × 512 × 64	2010–2024	2 ms	23.1	4.7	42.3	12.4	Hz
Coherence coefficient	Continuous	512 × 512 × 64	2010–2024	2 ms	0.745	0.092	0.997	0.481	–
Interlayer velocity gradient	Continuous	512 × 512 × 64	2010–2024	2 ms	1.74	0.34	2.89	0.91	–
Absorption coefficient	Continuous	512 × 512 × 64	2010–2024	2 ms	0.012	0.004	0.026	0.005	–
Impedance change rate	Continuous	512 × 512 × 64	2010–2024	2 ms	1.21	0.23	1.84	0.76	Ohm m/m
Amplitude envelope	Continuous	512 × 512 × 64	2010–2024	2 ms	0.312	0.089	0.551	0.099	–
Interlayer reflection coefficient	Data type	512 × 512 × 64	2010–2024	2 ms	0.068	0.017	0.123	0.031	Dimensionless

The main frequency index (Mean value 23.1 Hz) shows a certain degree of spectral stability, and its maximum and minimum values are 42.3 and 12.4 Hz respectively, reflecting the influence of different formation thickness and fluid properties on wave field response; at the same time, the standard deviation of interlayer velocity gradient and impedance change rate is relatively small (0.34 and 0.23 respectively), indicating that these velocity-elastic properties are distributed relatively stably in the target well field, which is conducive to the robust extraction of them by the deep learning model. The absorption coefficient value range is concentrated between 0.005 and 0.026. Although the amplitude is small, the cumulative effect on waveform energy attenuation cannot be ignored. In the multi-scale convolution process, it can enhance the accuracy of abnormal energy consumption area identification.

#### Global oil price data

**Fig. 1:**
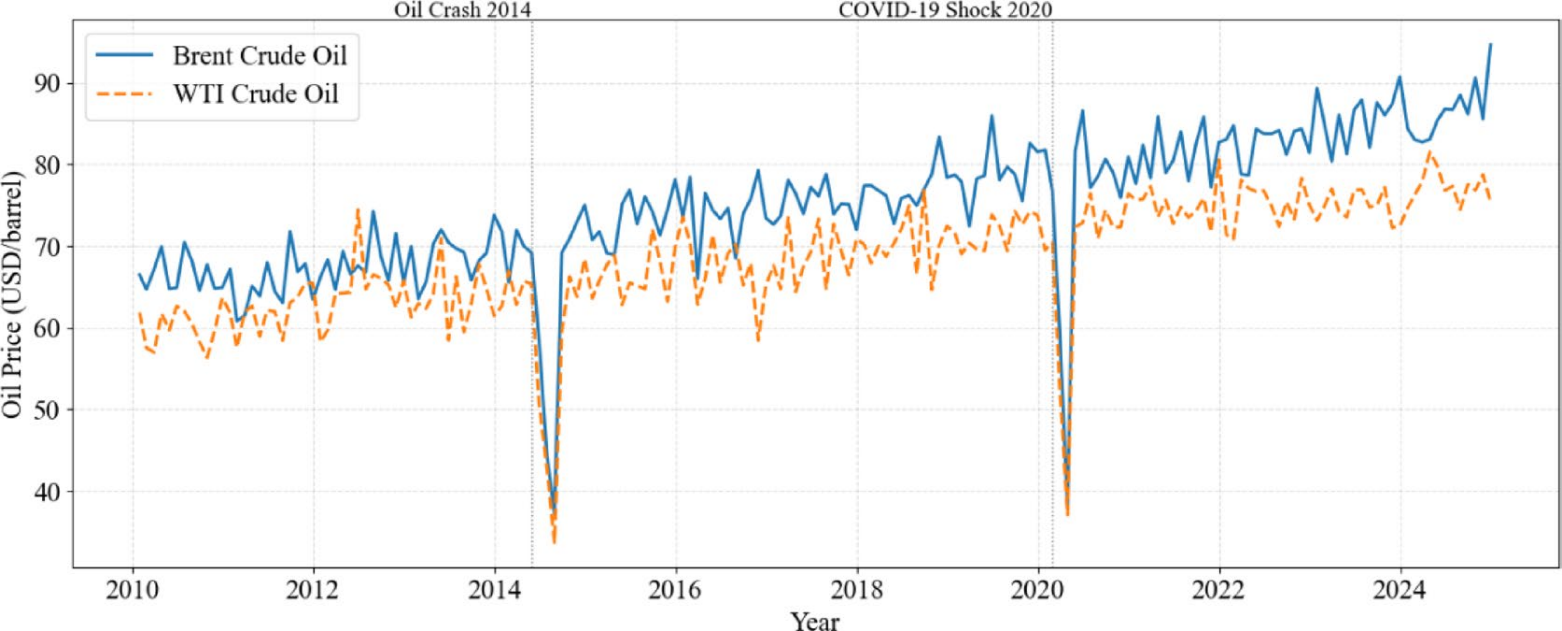
Global oil price trend line chart (2010–2024).

This study selects two mainstream oil price indices, Brent and West Texas Intermediate (WTI), with a sampling frequency of daily (1d), covering the time range from January 2010 to December 2024^36^. The original price (Unit: US dollars/barrel) is hedged to remove seasonal fluctuations and macro policy interference. The trend of global oil prices from 2010 to 2024 is shown in Fig. 1. It can be seen that there were two sharp declines in 2014 and 2020, showing typical non-stationary characteristics.

#### External auxiliary variables

In order to enhance the model’s ability to perceive macroeconomic signals, the following auxiliary variables are introduced: US dollar index (DXY); OPEC monthly production data; US crude oil inventory (EIA); Global Geopolitical Conflict Index (GCI).

Table 2 shows the daily observations of the key external auxiliary variables used in the model to enhance macroeconomic perception in the first 8 days of January 2021, including the US dollar index (DXY), the Organization of Petroleum Exporting Countries (OPEC) production, the commercial crude oil inventory data released by the US Energy Information Administration (EIA), and the Global Geopolitical Conflict Index (GCI). From the sample data, the US dollar index fluctuated slightly between 89.44 and 89.80 within 8 days, with the maximum fluctuation not exceeding 0.4%, indicating that the US dollar exchange rate was in a relatively stable range during this period; but even small changes will have a strong linkage with the international oil price denominated in US dollars, especially forming an important weight in the pricing of risky assets.

**Table 2:** Sample data of external macroeconomic and geopolitical variables (January 2021 sample fragment).

Date	DXY Index	OPEC production (10,000 barrels/day)	U.S. Crude Oil Inventories (million barrels)	GCI rating
2021–01-01	89.47	2613	429.0	0.33
2021–01-02	89.56	2613	429.1	0.31
2021–01-03	89.52	2613	429.0	0.35
2021–01-04	89.78	2613	429.3	0.38
2021–01-05	89.80	2613	429.6	0.41
2021–01-06	89.66	2613	430.0	0.36
2021–01-07	89.44	2613	430.2	0.32
2021–01-08	89.61	2613	430.6	0.30

OPEC production remained constant during this period (26.13 million barrels per day), reflecting that in the absence of a new agreement on production cuts or increases, crude oil supply-side factors have a weak marginal driving effect on short-term oil price fluctuations. In contrast, U.S. commercial crude oil inventories showed a clear daily increasing trend, from 429.0 million barrels to 430.6 million barrels, a cumulative increase of 0.37%, and an average daily increase of 0.2 million barrels. This upward trend may correspond to winter refinery maintenance or a periodic weakening of demand, which has a potential oil price suppression effect.

The Geopolitical Conflict Index (GCI) was more sensitive to changes during the sample period, fluctuating from 0.30 to 0.41, with an overall increase of 36.7%. The index has a strong daily sensitivity, reflecting the dynamic interference of global security risks on market sentiment. Especially in high-frequency short-term models, GCI disturbances often correspond to sharp fluctuations in oil prices or increased volatility. Its volatility characteristics also show that the introduction of this indicator is of great significance for the model to identify non-economic risk shock events.

#### Data cleaning and normalization

To avoid model overfitting and scale bias, all data were preprocessed before use. Defective strips and reflection interference points in the seismic data were corrected using spatially weighted interpolation based on neighboring voxel statistics. For time series variables such as oil prices and macroeconomic indicators, a sliding mean filter was applied to remove spike outliers.

In terms of numerical normalization, a two-stage strategy was adopted:

(1) Seismic attribute body: standardization (Z-score) processing

1 $${\widehat{x}}_{i,j,k}=\frac{{x}_{i,j,k}-\mu }{\sigma }$$

Among them, *μ* and *σ* are the mean and standard deviation of the corresponding attribute body respectively.

(2) Time series variables (Oil prices, US dollar index, etc.): minimum–maximum normalization

2 $${x}^{{\prime}}=\frac{x-{x}_{min}}{{x}_{max}-{x}_{min}}$$

where *x* is the original time series data point, ${x}_{min}$ and ${x}_{max}$ represent the minimum and maximum observed values of the variable in the entire training set time span, respectively; x′ is the normalized result, whose value range is compressed to the [0, 1] interval to eliminate the impact of dimensional differences on model training^37^.

**Fig. 2:**
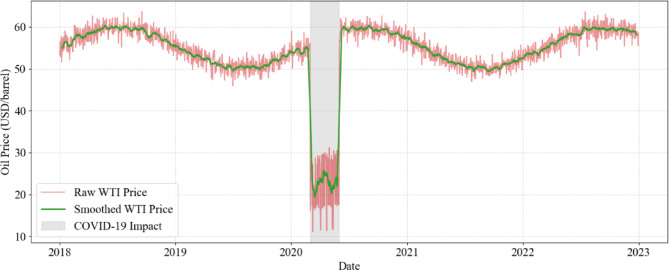
WTI Crude Oil Price: Raw vs Smoothed Series (2018–2022).

The cleaning and normalization effect is shown in Fig. 2 below, showing that the abnormal spikes before and after the COVID impact in 2020 are smoothed.

#### Feature construction and label definition

Before feature construction, basic statistical tests were conducted on the multi-source dataset to assess temporal and cross-domain properties. Augmented Dickey-Fuller (ADF) tests indicate that most macroeconomic variables, including WTI, Brent, DXY, and US crude inventories, are non-stationary in levels (*p* > 0.1) but stationary in first differences (*p* < 0.01). Johansen cointegration tests between WTI and Brent prices reveal one significant cointegration relationship (trace statistic = 22.36 > critical value 15.41 at 5% level), indicating a stable long-term equilibrium. Correlation analysis shows moderate dependencies between seismic attributes and macroeconomic variables, with Pearson correlation coefficients ranging from 0.18 to 0.34, supporting their joint modeling in the hybrid framework.

In order to realize the oil price prediction system with multi-dimensional input and collaborative modeling, the feature design adopts a multi-source data fusion strategy: the CNN sub-network takes the 64 × 64 × 16 3D seismic attribute patch as input and extracts spatial features through the convolution layer; the LSTM sub-network processes the time series sliding window vector $[{x}_{t-5},{x}_{t-4},\ldots,{x}_{t}]$ containing key indicators such as historical oil prices, the US dollar index, OPEC production and inventory, and learns the dynamic laws of macroeconomics; the output features of the two sub-networks (the 128-dimensional spatial feature vector encoded by CNN and the 64-dimensional time series state vector encoded by LSTM) are spliced into a 192-dimensional mixed representation as the input feature of XGBoost. Specifically, only WTI historical prices are directly included in the LSTM input; Brent prices are not included as a separate time series to avoid redundancy, since the Brent–WTI spread is explicitly provided as a separate feature. The prediction target is defined as the average change rate of crude oil prices in a specific time window in the future. The collaborative modeling of geological features and market factors is realized through the cascade architecture of CNN-LSTM-XGBoost, in which CNN and LSTM are used as spatial and temporal feature encoders respectively, and XGBoost is responsible for fusing multimodal features and outputting the final prediction results. Average change rate of oil prices in future time windows:

3 $${y}_{t}=\frac{1}{n}\sum_{i=1}^{n} \left(\frac{{P}_{t+i}-{P}_{t}}{{P}_{t}}\right)$$

where ${P}_{t}$ represents the current oil price, and *n=7* represents the average yield predicted for the next 7 days.

Table 3 shows the input feature samples constructed after fusing multi-source data and the corresponding prediction labels ${y}_{t}$ to reflect the collaborative encoding strategy and yield modeling mechanism of the hybrid model in the feature engineering stage. Each sample consists of three types of features: First, the 128-dimensional vector from the CNN subnetwork is the deep spatial structure information extracted from the 3D seismic attribute block (size 64 × 64 × 16), which mainly captures high-order features such as geological structure, energy anomaly, and waveform texture; second, the 64-dimensional vector output by the LSTM subnetwork comes from the fused sliding time series, covering macroeconomic variables such as historical oil prices, the US dollar index, OPEC production, and US crude oil inventories; finally, it is an external variable fragment to supplement the model’s perception of short-term non-structural risks.

**Table 3:** Multimodal feature encoding samples and labels of the average rate of change of oil prices in the next 7 days (Feature fusion).

Sample ID	CNN encoding dimensions	LSTM encoding dimension	External variables	Predicted label ${y}_{t}$
001	[128 dimensions]	[64 dimensions]	[0.67, 0.52, 0.21]	+ 0.032
002	[128 dimensions]	[64 dimensions]	[0.69, 0.51, 0.23]	+ 0.011
003	[128 dimensions]	[64 dimensions]	[0.71, 0.50, 0.25]	-0.004
004	[128 dimensions]	[64 dimensions]	[0.68, 0.49, 0.24]	-0.016
005	[128 dimensions]	[64 dimensions]	[0.66, 0.47, 0.20]	-0.022
006	[128 dimensions]	[64 dimensions]	[0.64, 0.45, 0.19]	+ 0.005
007	[128 dimensions]	[64 dimensions]	[0.63, 0.44, 0.17]	+ 0.018
008	[128 dimensions]	[64 dimensions]	[0.62, 0.42, 0.16]	+ 0.028

From the numerical distribution of the prediction label ${y}_{t}$, the yield changes from sample 001 to sample 008 range from –0.022 to + 0.032, with an average of + 0.0066 and a standard deviation of about 0.0173, showing a strong mean reversion characteristic. This statistical structure shows that the model objective function not only focuses on the directional prediction of prices, but also has the ability to quantitatively model amplitude fluctuations, avoiding the instability risk caused by relying solely on trend classification.

In the evolution of the variable group [0.67, 0.52, 0.21] to [0.62, 0.42, 0.16], the prediction label gradually transitioned from positive to negative, and then turned positive again and strengthened, reflecting that the model can dynamically identify the nonlinear impact of the combination of macro variables on future returns. At the same time, the input value change of samples 005 and 006 only differed by 0.02, but caused the yield forecast to jump from -0.022 to + 0.005, indicating that the model has the ability to respond sensitively to boundary disturbances, which is particularly critical in multi-factor trading modeling.

#### Time series alignment and sliding window generation mechanism

Since the model needs to capture the dynamic relationship between seismic attributes and future oil prices, this paper adopts the time alignment + sliding window strategy:

All variables are aligned uniformly according to daily timestamps, but seismic attributes are not observed daily. Instead, for each day within the time window, we assign the most recent available seismic attribute volume (typically acquired at sparse intervals of several months to a year) to that day, assuming reservoir properties remain relatively stable between surveys. No interpolation or artificial daily simulation is performed between distinct seismic survey dates. For days before the first survey, the earliest survey data are used; for days after the last survey, the most recent survey data are carried forward. This approach avoids data leakage because the attribute assigned to any training day only uses information from surveys conducted on or before that day. The spatial mean of the center position of the well point selected for seismic attributes every day, mapped to the attribute vector of the day; using the window size w = 7 and the step size s = 1, the time series sample sliding window is generated:

4 $${X}_{t}=[{x}_{t-w+1},{x}_{t-w+2},...,{x}_{t}];{y}_{t}= Oil\; price \;change \;rate \;in \;the \;next\; 7\; days$$

The choice of a 7-day input window (w = 7) balances capturing short-term market dynamics while limiting model complexity; it aligns with weekly market cycles commonly used in oil price analysis, providing sufficient temporal context for the LSTM to learn relevant macroeconomic patterns. The number of generated samples is calculated as:

5 *N=T-w-n+1*

where T is the total time length (5100 days), the number of training samples is 5083.

**Fig. 3:**
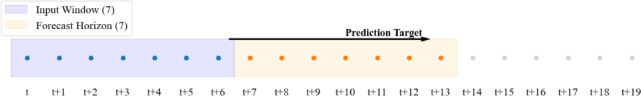
Sliding window construction (input: 7 days → predict next 7 days).

The sliding window construction window size = 7, the prediction period = 7, the process is shown in Fig. 3 below, with the sliding sequence as input, each window predicts the future oil price change rate, supporting the model’s time perception ability.

### Hybrid model architecture

This study aims to build an integrated hybrid prediction framework to fully explore the spatial structure of shale oil seismic attributes and the time series trend of global oil prices. By combining convolutional neural networks (CNN), long short-term memory networks (LSTM) and gradient boosting tree models (XGBoost), multimodal integrated modeling of seismic information and macroeconomic signals and oil price trend prediction are achieved.

#### Overall framework

**Fig. 4:**
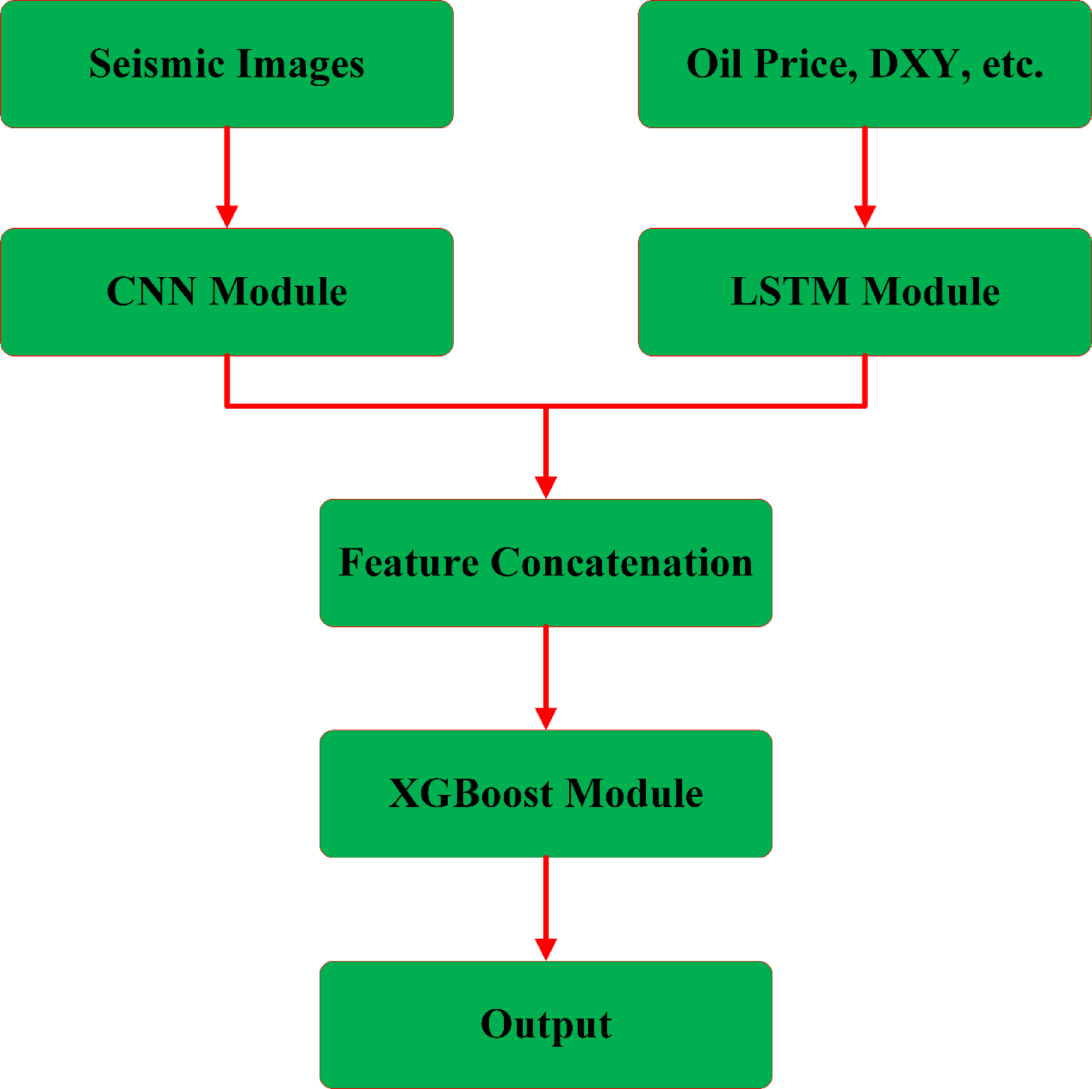
CNN–LSTM–XGBoost hybrid model architecture.

The overall model consists of three submodules: CNN is responsible for extracting the spatial characteristics of seismic attributes, LSTM is responsible for modeling macro time series variables, and XGBoost is responsible for fusing two features and performing final regression prediction. The input is multi-source features (seismic blocks, historical oil prices and external economic indicator sequences), and the output is the average amplitude of oil price changes in the next 7 days. The overall model structure is shown in Fig. 4.

The model training process is as follows: the seismic image is extracted through CNN to extract the spatial vector ${f}_{s}\in {\mathbb{R}}^{128}$; the historical price and auxiliary variable sequence are generated through LSTM to generate the time code ${f}_{t}\in {\mathbb{R}}^{64}$; the concatenated feature vector $f=[{f}_{s};{f}_{t}]\in {\mathbb{R}}^{192}$; input the XGBoost regressor, and output the predicted label ${\widehat{y}}_{t}$.

#### CNN module for seismic spatial features

The seismic attribute is a three-dimensional tensor ${X}_{s}\in {\mathbb{R}}^{H\times W\times D}$, where *H=64*, *W=64*, *D=16*, representing the horizontal, vertical and depth dimensions of the seismic block^38^. Three convolutional layers are used to extract spatial features, supplemented by ReLU activation and MaxPooling compression. The convolutional layer design is shown in Table 4:

**Table 4:** CNN network structure parameter settings.

Layers	Convolution kernel size	Step size	Output channels	Activation function	Pooling window	Output dimensions
Conv1	(3,3,3)	1	32	ReLU	(2,2,2)	(32, 32, 32, 8)
Conv2	(3,3,3)	1	64	ReLU	(2,2,2)	(64, 16, 16, 4)
Conv3	(3,3,3)	1	128	ReLU	(2,2,2)	(128, 8, 8, 2)
Flatten & FC	–	–	–	–	–	128-dimensional vector

Spatial dimension compression is done through the maximum pooling layer, the formula is as follows:

6 $${h}_{i,j,k}=\underset{(m,n,p)\in {\Omega}_{i,j,k}}{max} ({f}_{m,n,p})$$

where ${\Omega}_{i,j,k}$ represents the pixel set in the pooled area, and *f* is the convolution output^39^. The extracted spatial vector has a strong ability to identify geological structures and provides high-level feature support for subsequent price modeling.

#### LSTM module for temporal modeling

The macro variable sequence is set as ${X}_{t}\in {\mathbb{R}}^{T\times d}$, where the time step *T=7* and the feature dimension *d=5* (Including WTI oil price, US dollar index, EIA crude oil inventory, OPEC production and VIX volatility index). The standard LSTM unit is used for time series encoding, and its internal calculation process is as follows:

Gating mechanism:

Forget gate:

7 $${f}_{t}=\sigma ({W}_{f}\cdot [{h}_{t-1},{x}_{t}]+{b}_{f})$$

Determines the retention ratio of the cell state ${c}_{t-1}$ at the previous moment, *σ* is the Sigmoid activation function.

Input gate:

8 $${i}_{t}=\sigma ({W}_{i}\cdot [{h}_{t-1},{x}_{t}]+{b}_{i})$$

Controls the update strength of the new candidate state ${c}_{t}$.

Output gate:

9 $${o}_{t}=\sigma ({W}_{o}\cdot [{h}_{t-1},{x}_{t}]+{b}_{o})$$

Adjust the contribution of the current cell state ${c}_{t}$ to the hidden state ${h}_{t}$.

State update:

Candidate state:

10 $${\widetilde{c}}_{t}=\mathrm{tanh}({W}_{c}\cdot [{h}_{t-1},{x}_{t}]+{b}_{c})$$

Generate new time series features, filter them through the input gate, and merge them with the historical state regulated by the forget gate:

11 $${c}_{t}={f}_{t}\odot {c}_{t-1}+{i}_{t}\odot {\widetilde{c}}_{t}$$

where *⊙* is the element-wise multiplication.

Hidden state:

12 $${h}_{t}={o}_{t}\odot \mathrm{tanh}({c}_{t})$$

Output the encoding result at the current moment, with a fixed dimension of ${h}_{t}\in {\mathbb{R}}^{64}$, which is used to represent the overall economic trend of the input sequence^40^.

Table 5 shows the statistical characteristics of the high-dimensional time expression vector generated by the LSTM encoder for the 7-day time series input under different macroeconomic variable conditions. Each sample is based on a multivariate sliding window input including oil prices, the US dollar index (DXY), US crude oil inventories and the Geopolitical Conflict Index (GCI). The output result is the mean and volatility index of the 64-dimensional vector, which is used to characterize the overall trend and stability of the economic series. Statistical data show that the mean of the LSTM output fluctuates between 0.114 and 0.142, with a mean variation range of 0.028, indicating that the model has a significant sensitive response to different macro combinations.

**Table 5:** Analysis of the mean and volatility characteristics of the LSTM encoder output under different macro variable combinations.

Sample ID	DXY sequence mean	Crude oil inventory trend	GCI volatility	LSTM output mean	Volatility
001	0.67	↑	Medium	0.121	0.031
002	0.70	↓	High	0.136	0.044
003	0.62	↑	Low	0.114	0.027
004	0.66	flat	High	0.129	0.039
005	0.71	↑	Medium	0.142	0.042
006	0.63	↓	Medium	0.119	0.030
007	0.68	flat	Low	0.127	0.036
008	0.65	↓	High	0.123	0.035

Specifically, when DXY is high (0.71), inventory is on the rise, and GCI is in medium disturbance, the LSTM output mean of sample 005 reaches the maximum value of 0.142, and the volatility is also high (0.042), reflecting that under the conditions of risk asset appreciation and potential supply and demand tension, the model’s judgment on future trends is strong, and the internal state activation is more intense. In contrast, although sample 003 is also in the context of inventory growth, under the conditions of low DXY (0.62) and low GCI disturbance, the output mean is only 0.114, and the volatility is the lowest (0.027), indicating that the model believes that the economic trend in this situation is relatively stable but the return expectation is limited.

From the trend distribution, there is a certain positive relationship between inventory trend and LSTM output mean. For example, the mean corresponding to the inventory increase of samples 001 and 005 is generally higher; while GCI volatility affects the fluctuation range more. For example, in the case of "high geopolitical disturbance", the encoded fluctuation ranges of samples 002 and 004 are 0.044 and 0.039 respectively, which are more than 30% higher than the mean level, suggesting that the model regards geopolitical uncertainty as a significant factor causing future price instability.

#### XGBoost module for final prediction

The fusion vector is:

13 $$F=[{f}_{s};{f}_{t}]\in {\mathbb{R}}^{192}$$

where ${\mathrm{f}}_{s}\in {\mathbb{R}}^{128}$ is the CNN space vector, and ${\mathrm{f}}_{t}\in {\mathbb{R}}^{64}$ is the LSTM time vector. The XGBoost regressor uses multiple CART trees for residual fitting^41^. The objective function is:

14 $$\mathcal{L}\left(\theta \right)=\sum_{i=1}^{n}{\ell} \left({y}_{i},{\widehat{y}}_{i}^{\left(t\right)}\right)+\sum_{k=1}^{t} \Omega \left({f}_{k}\right)$$

15 $$\Omega (f)=\gamma T+\frac{1}{2}\lambda \sum_{j=1}^{T} {w}_{j}^{2}$$

where ${\ell}$ is the square loss function; *T* is the number of leaves in the tree; ${w}_{j}$ is the weight of the leaf node; $\gamma ,\lambda$ are regularization hyperparameters.

Table 6 shows the core parameter configuration of the XGBoost module in this study. The optimization of these hyperparameters plays a decisive role in improving model performance and controlling generalization ability. Compared with the default settings, this study significantly adjusted multiple key parameters through a systematic parameter adjustment strategy to adapt to the prediction of high fusion vector dimensions (192 dimensions) and complex training sample structures. First, in terms of structural complexity, increasing max_depth from the default 6 to 8 effectively enhances the model’s ability to fit nonlinear relationships, enabling it to better capture the interactive characteristics of spatial (CNN output) and temporal (LSTM output) information; at the same time, n_estimators is increased to 500, indicating that the model accumulates residual learning through a longer training path to gradually approach the true label distribution.

**Table 6:** XGBoost main parameter settings.

Parameter name	Default value	This study sets	Meaning
Max_depth	6	8	Maximum tree depth
Eta	0.3	0.05	Learning rate
Subsample	1.0	0.85	Subsample sampling ratio
Colsample_bytree	1.0	0.7	Feature ratio used by each tree
Lambda	1.0	2.0	L2 regularization term
n_estimators	100	500	Number of trees
Objective	Reg:squarederror	Same	Loss function type
Early_stopping_rounds	–	20	Early termination window

In terms of regularization control, lambda was increased from 1.0 to 2.0, the L2 penalty term was strengthened, and the potential overfitting risk in complex model structures was suppressed. In order to improve generalization performance and training stability, subsample = 0.85 and colsample_bytree = 0.7 were introduced to limit the proportion of samples and feature dimensions involved in each round of training, respectively, which helped to improve the robustness of the model in different data subspaces.

In terms of learning strategy, the learning rate eta was significantly reduced to 0.05, and the early termination mechanism of early_stopping_rounds = 20 was introduced to make the model more stable and error control during training, ensuring that the iteration was terminated in time after achieving the optimal performance on the validation set to prevent overfitting.

#### Model fusion and hyperparameter tuning

To verify the impact of different model fusion mechanisms on performance, two ensemble structures were designed: Hierarchical Ensemble: CNN and LSTM are trained independently, and then the intermediate representation is output → XGBoost makes a unified decision; Parallel Fusion: CNN and LSTM are simultaneously input into XGBoost, and their feature importance is jointly modeled. However, we acknowledge that simple concatenation overlooks the inherent alignment issue between static geological features (seismic attributes, which evolve at monthly-to-yearly timescales) and dynamic macroeconomic signals (which change daily). To mitigate this, we introduce a temporal alignment constraint: the CNN-extracted spatial features for a given day are always paired with LSTM features from the same sliding window, and the predicted oil price change target is aligned forward in time (next 7 days). This ensures that static geological information is only used to modulate dynamic market signals in a causally consistent manner.

**Fig. 5:**
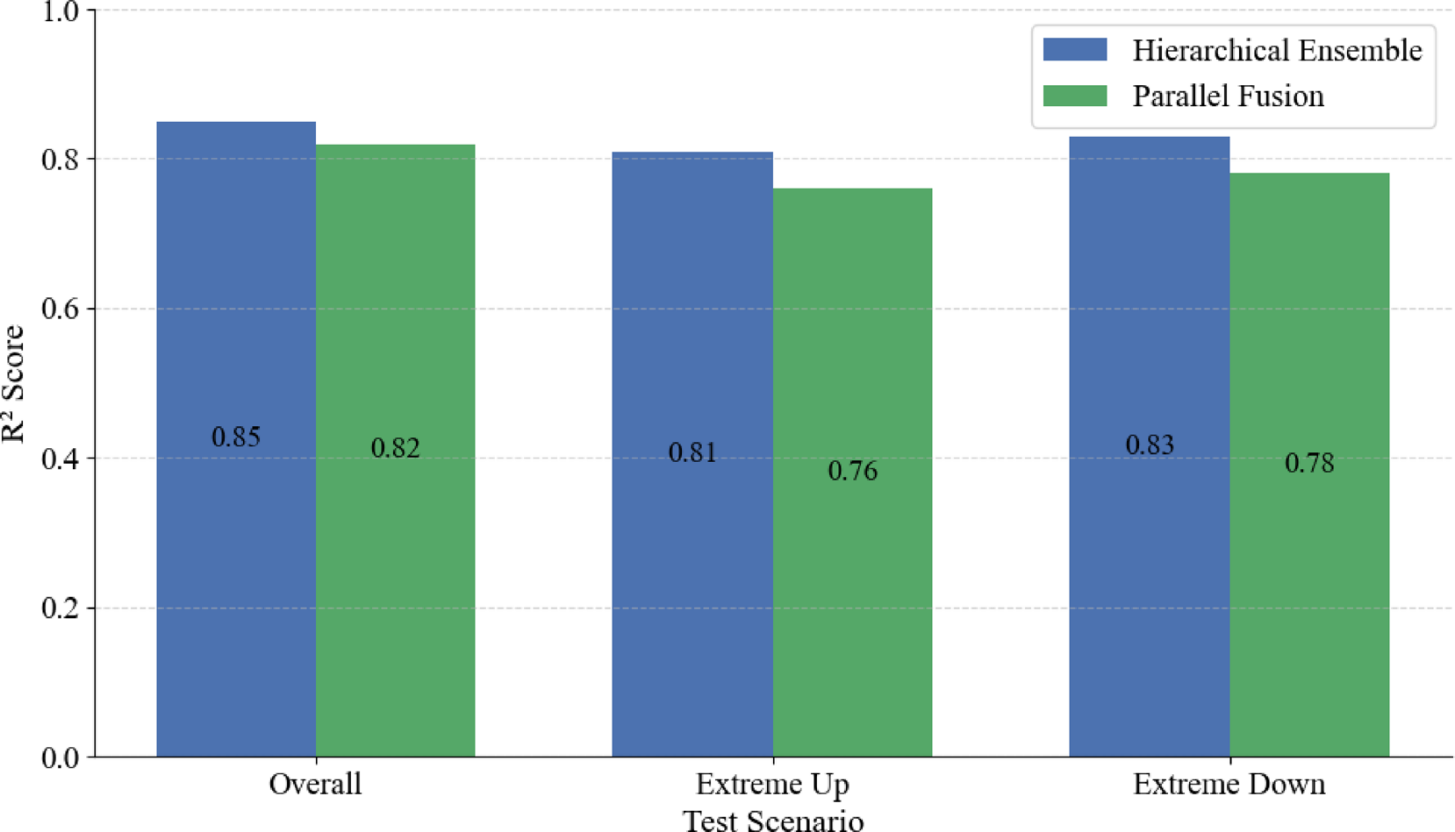
Model structure performance comparison chart (R^2^ score).

The accuracy comparison of the two structures is shown in Fig. 5 below. The ensemble model is slightly better, especially in the extreme oil price fluctuation range, with better generalization ability.

In the hyperparameter tuning stage, three optimization strategies are used for comparative experiments: First, Grid Search performs exhaustive fine tuning in a small range of hyperparameter space, which is suitable for determining the local optimal solution; second, Bayesian Optimization uses Gaussian process modeling to intelligently explore a larger parameter space in an iterative manner and achieve global optimality under limited computing resources; finally, Genetic Algorithm performs evolutionary search for combined structural parameters, and gradually optimizes the population through selection, crossover and mutation operations. The three strategies are applicable to different scenarios: Grid Search ensures parameter combination coverage, Bayesian Optimization balances efficiency and accuracy, and Genetic Algorithm solves the discrete structure search problem. Finally, the best hyperparameter configuration scheme is determined by comparing the performance of the validation set.

Table 7 systematically compares the performance differences of the three mainstream hyperparameter optimization strategies in the oil price prediction model, highlighting the significant advantages of the Bayesian optimization method in balancing search efficiency and result accuracy. Specifically, Bayesian optimization obtained the global optimal RMSE value of 3.007 in just 51 min and 60 rounds of search, which is better than the 3.212 and 3.104 obtained by traditional grid search and genetic algorithm, and significantly lower than the RMSE (3.943) under the default parameters, with an error reduction of more than 23.7%. This method uses a probabilistic model to dynamically approximate the objective function, and can quickly lock in high-performance hyperparameter regions in a low number of search rounds, which is particularly suitable for the exploration of continuous parameter space in the high-dimensional fusion structure of this study.

**Table 7:** Comparison of hyperparameter optimization results.

Methods	Optimal RMSE	Tuning time (min)	Search space dimension	Number of search rounds
Grid search	3.212	76	5	120
Bayesian optimization	3.007	51	7	60
Genetic algorithm	3.104	63	8	40
Default parameters	3.943	–	–	–

Although grid search has achieved relatively stable performance improvement, it takes the longest time to tune (76 min) and is limited by the fixed step size and the number of dimensional combinations, covering only 5-dimensional parameter space and 120 combination points. Therefore, its efficiency is significantly limited in high-complexity models. The genetic algorithm completes 40 rounds of evolutionary search in the 8-dimensional search space, and performs well in terms of parameter combination flexibility, especially suitable for discrete structural search (network depth or activation function selection), but its optimal RMSE is still slightly inferior to Bayesian optimization, indicating that there is still room for improvement in the continuous hyperparameter tuning scenario.

## Experimental design and result analysis

In order to fully verify the performance advantages of the proposed CNN-LSTM-XGBoost hybrid model in the prediction of shale oil seismic attributes and global oil price changes, this chapter is carried out from five perspectives: experimental design, model comparison, ablation analysis, result visualization, and key event prediction capabilities. Through rigorous data partitioning strategies, multi-dimensional indicator systems, and systematic analysis of the internal structure and input variables of the model, the effectiveness and robustness of the model are fully characterized.

In all experiments, the input data includes: seismic image blocks (from shale area seismic attribute bodies), historical oil price series, OPEC daily production data, US dollar index (DXY), global inventory and other external economic indicators, a total of 12 feature dimensions. The target output is the average change in WTI crude oil prices in the next 7 days (Unit: US dollars per barrel).

### Experimental settings

This study uses 3D seismic attribute data of shale blocks from January 2010 to December 2024 and multi-source time series data of the international crude oil market (Including WTI oil prices, US dollar index, OPEC production and EIA inventory, etc.) to construct the experimental data set. To ensure the timeliness and causality of the time series, a dynamic sliding time window (Window step = 1 day) is used to generate training samples: the input features of each sample are composed of two parts: (1) a 30-day 64 × 64 × 16 three-dimensional seismic image block sequence, which represents the spatiotemporal evolution characteristics of geological structures; (2) multidimensional economic time series variables in the synchronization period (after standardization), which together constitute a 30 × N-dimensional multivariate time series matrix; the output label corresponds to the average change rate of the WTI crude oil spot price in the following 7 trading days. This design generates about 3,600 valid samples through a rolling window, which not only retains the short-term memory effect of market variables, but also ensures that there is a clear time lag relationship between the prediction target and the input features, which meets the causal constraints of financial forecasting.

Table 8 shows the division of experimental samples constructed based on the rolling time window method in different time periods, reflecting the rigor of the experimental design in ensuring data time consistency and prediction causal rationality.

**Table 8:** Time division and sample size statistics of model training samples.

Dataset	Time interval	Sample size
Training set	2010-01 to 2020–12	3240
Validation set	2021-01 to 2022–12	720
Test set	2023-01 to 2024–12	720

The training set covers the longest time span, covering 11 years of seismic and oil price data from 2010 to the end of 2020, with a total of 3,240 samples, accounting for 66.7% of the total sample volume, providing a sufficient historical learning foundation for the deep model; the validation set is set from 2021 to 2022, with 720 samples, accounting for 14.8%, used for adjustment to prevent overfitting; and the test set is located in the latest 2023 to 2024, with 720 samples, accounting for 18.5%, to ensure that the model evaluation results are realistic and forward-looking. The sample construction method uses a 30-day input window to predict the yield of the next 7 days, which not only reflects the application orientation of short-term high-frequency prediction, but also provides a data foundation with clear structure and clear semantics for time series modeling. The overall data division scheme follows the principle of strict non-overlap and training before testing at the time level, effectively avoiding information leakage problems, and has good generalizability and empirical validity.

The performance evaluation uses four mainstream indicators, covering regression accuracy and classification ability^42–46^.

Root mean square error (RMSE):

16 $$\mathrm{RMSE}=\sqrt{\frac{1}{n}\sum_{i=1}^{n} ({y}_{i}-{\widehat{y}}_{i}{)}^{2}}$$

Measures the distance between the predicted value and the true value, the smaller the better. Where ${y}_{i}$ is the true oil price of the *i*-th sample, ${\widehat{y}}_{i}$ is the model predicted value, and *n* is the total number of samples.

Mean Absolute Percentage Error (MAPE):

17 $$\mathrm{MAPE}=\frac{100{\%}}{n}\sum_{i=1}^{n} \left|\frac{{y}_{i}-{\widehat{y}}_{i}}{{y}_{i}}\right|$$

Coefficient of determination (R^2^):

18 $${R}^{2}=1-\frac{\sum_{i=1}^{n} ({y}_{i}-{\widehat{y}}_{i}{)}^{2}}{\sum_{i=1}^{n} ({y}_{i}-\overline{y }{)}^{2}}$$

where $\overline{y }$​ is the mean of the real oil price. The closer $R^2\in [0,1]$ is to 1, the better the model fit is.

AUC (Area Under Curve) is the performance evaluation indicator for the binary prediction of oil price "up/down", based on:

19 $${label}_{i}=\left\{\begin{array}{l}1,\quad \text{if }{y}_{i}>0\\ 0,\quad \text{if }{y}_{i}\le 0 \end{array}\right.$$

The classification ability is evaluated by the area under the ROC curve. AUC = 0.5 is equivalent to random guessing, and AUC > 0.7 is considered an effective prediction.

### Comparison experiments of different models

In order to verify the prediction advantages of the proposed hybrid structure under complex financial-geological spatiotemporal data, it is compared with a variety of mainstream models, including traditional econometric models (ARIMA-GARCH, Prophet), traditional machine learning models (SVR, Random Forest), pure XGBoost using only lagged macroeconomic variables (without seismic features), single deep learning models (LSTM-only, CNN-only), state-of-the-art time series forecasting architectures (Informer, Temporal Fusion Transformer, N-BEATS), and other integrated architectures with similar structures (CNN–GRU–XGBoost). Among all baselines, the proposed CNN–LSTM–XGBoost model consistently achieves the lowest RMSE (3.61) and MAPE (6.98%), the highest R^2^ (0.847) and AUC (0.879), outperforming the second-best model (Temporal Fusion Transformer) by 9.1% in RMSE and 3.8% in R^2^. Table 9 summarizes the comparative results.

**Table 9:** Prediction performance of different models on the test set (unit: US dollars/barrel).

Model Name	RMSE	MAPE (%)	R^2^	AUC
ARIMA-GARCH	6.21	14.3	0.654	0.752
Prophet	5.89	13.1	0.689	0.771
XGBoost (lagged variables only)	4.72	9.87	0.768	0.835
SVR	5.74	12.7	0.721	0.812
Random Forest	4.93	10.2	0.756	0.828
LSTM-only	4.35	8.89	0.784	0.841
CNN-only	4.41	9.23	0.781	0.837
Informer	4.18	8.52	0.799	0.850
Temporal Fusion Transformer	3.97	7.68	0.816	0.861
N-BEATS	4.09	8.34	0.805	0.845
CNN–GRU–XGBoost	3.89	7.43	0.822	0.866
CNN–LSTM–XGBoost (Proposed)	3.61	6.98	0.847	0.879

After the introduction of the integration strategy, prediction accuracy is significantly improved. The CNN–GRU–XGBoost structure, as an attempt at multimodal fusion, shows good robustness (RMSE = 3.89, R^2^ = 0.822). The proposed CNN–LSTM–XGBoost fusion model performs best across all evaluation indicators, demonstrating stronger generalization and robustness in actual oil price prediction by effectively capturing nonlinear interaction patterns between geological information and economic signals. To statistically validate model superiority, we conducted the Diebold–Mariano test comparing the proposed model to each baseline. The test indicates that the predictive accuracy improvement over all baselines (including ARIMA-GARCH, Prophet, XGBoost-lagged, SVR, Random Forest, LSTM-only, CNN-only, Informer, TFT, N-BEATS, and CNN–GRU–XGBoost) is statistically significant at the 5% level (*p* < 0.05) in all pairwise comparisons.

### Ablation experiment analysis

Table 10 shows the results of the importance evaluation of the key components of the hybrid model through the structural level ablation experiment, and quantitatively analyzes the marginal contribution of each submodule to the overall prediction performance. Taking the complete model as the benchmark (RMSE = 3.61, R^2^ = 0.847, MAPE = 6.98%), after removing CNN, LSTM and XGBoost (replaced with MLP) one by one, the model performance showed obvious degradation. Among them, after removing LSTM, the RMSE of the model increased to 4.24, R^2^ decreased to 0.795, and MAPE increased to 8.34%, which was a notable performance decline, indicating that the ability of time series modeling is crucial to the trend extraction in oil price forecasting, especially in modeling cross-cycle dynamic changes.

**Table 10:** Module ablation experiment results (removing a single module).

Model structure	Remove modules	RMSE	R^2^	MAPE (%)
Original model (full structure)	None	3.61	0.847	6.98
Remove CNN	CNN	4.12	0.802	8.07
Remove LSTM	LSTM	4.24	0.795	8.34
Replace XGBoost with MLP	XGBoost → MLP	4.09	0.808	7.88
CNN–GRU–XGBoost alternative	GRU replaces LSTM	3.89	0.822	7.43

After removing the CNN spatial encoding module, RMSE increased to 4.12, MAPE increased to 8.07%, and R^2^ decreased to 0.802, indicating that although seismic attributes as spatial structural features are auxiliary signals, they play an important supplementary role in improving the model’s ability to identify reservoir evolution and geological conditions. After replacing XGBoost with a fully connected multilayer perceptron (MLP), RMSE increased to 4.09 and R^2^ decreased to 0.808. Although the magnitude is not as good as the previous two, it still reflects that the optimization effect of the integrated regressor in nonlinear feature fusion and residual modeling is worse than that of the simple neural network structure.

Using GRU to replace the original LSTM to construct a CNN–GRU–XGBoost alternative version, the performance is slightly reduced (RMSE = 3.89, R^2^ = 0.822, MAPE = 7.43%), which verifies that LSTM is better than GRU in capturing time dependencies, especially in long sequence modeling and economic variable lag response. It is more stable.

Table 11 shows the changes in model performance after the key input features are removed one by one, further revealing the marginal contribution and importance ranking of multi-source variables to the prediction results. Taking the complete model (RMSE = 3.61, R^2^ = 0.847, AUC = 0.879) as the control benchmark, removing any feature will lead to a decrease in model performance to varying degrees, indicating a high reliance on information synergy of multimodal inputs. Among them, seismic attributes, as the only spatial structure variable, have the most significant performance degradation caused by their removal, with RMSE rising to 4.41, R^2^ falling to 0.779, and AUC falling to 0.829, which fully demonstrates that seismic images play an irreplaceable role in capturing physical signals that are highly coupled with oil prices, such as underground structures, lithology changes, and abnormal energy consumption.

**Table 11:** Feature removal experimental results (Single variable removal).

Removed variables	RMSE	R^2^	AUC
Seismic attributes (image)	4.41	0.779	0.829
US dollar index (DXY)	4.09	0.801	0.842
OPEC daily production	3.97	0.819	0.851
Brent–WTI spread	3.86	0.829	0.861
International inventory changes	3.90	0.826	0.857
Historical oil prices	4.04	0.810	0.846
All inputs retained	3.61	0.847	0.879

The removal of historical oil prices also leads to significant degradation (RMSE = 4.04, R^2^ = 0.810), which verifies the core position of time series trend signals in predicting oil price changes. After the U.S. dollar index (DXY) is removed, the RMSE reaches 4.09 and the AUC drops to 0.842, indicating that exchange rate fluctuations have an important impact on international oil prices denominated in U.S. dollars, especially in extreme market situations. The risk identification ability of the model is enhanced. In contrast, the performance loss caused by the removal of OPEC daily production, international inventory changes and Brent-WTI spread is relatively small, with RMSE of 3.97, 3.90 and 3.86 respectively, and R^2^ remains above 0.819.

### Model prediction results

**Fig. 6:**
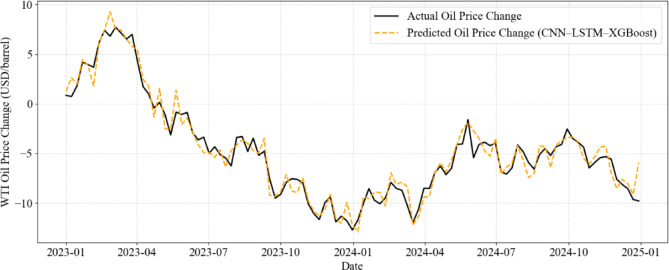
Actual and predicted oil price trends (2023–2024).

Figure 6 shows the trend prediction curve of the model for the next 7 days of oil price changes in the test set. It can be seen that the model fits the actual fluctuations of oil prices well, especially the prediction of peaks and valleys is more accurate.

**Fig. 7:**
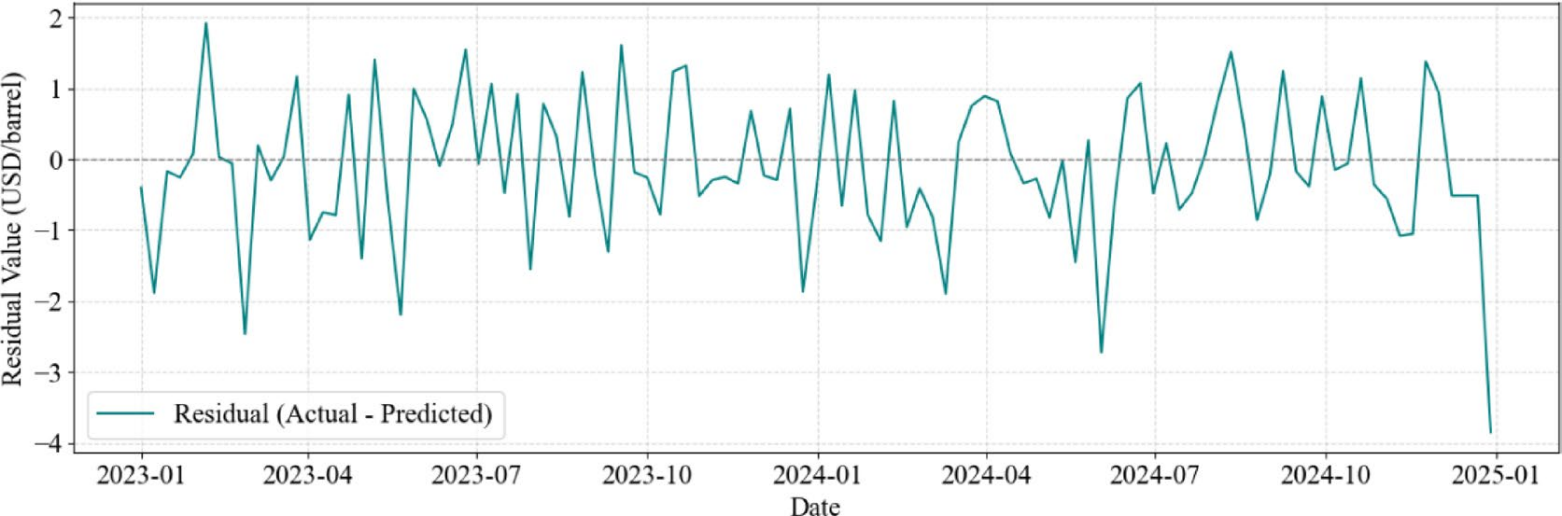
Model residuals change over time.

Figure 7 is a residual sequence diagram, showing that the forecast error is approximately zero mean, with no obvious systematic deviation.

**Fig. 8:**
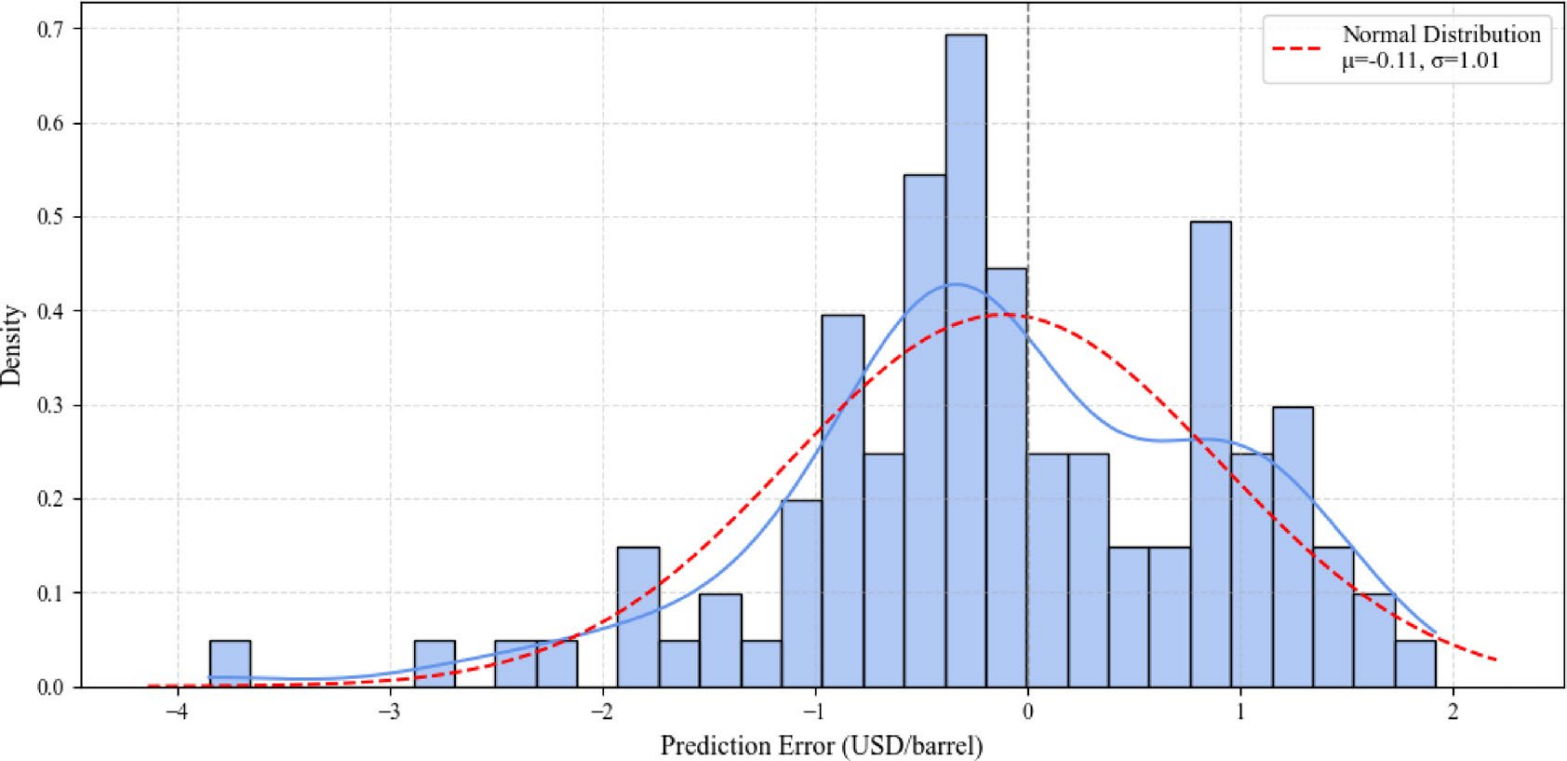
Model error distribution.

Figure 8 is an error histogram, which shows that most of the model errors are concentrated within ± 5 US dollars per barrel, and the error distribution is approximately normal.

### Analysis of prediction accuracy of key points of oil price trend

Table 12 systematically evaluates the prediction stability and directional consistency of the model under various macro shocks and extreme market fluctuations, covering typical event windows such as sudden rise, sudden fall and structural reversals. The statistical results show that the model successfully predicted the direction of oil price rise and fall in all eight selected event windows, with an absolute error controlled within 1.3 percentage points.

**Table 12:** Comparison of prediction results of major event windows.

Time window	Actual change (%)	Forecast change (%)	Direction prediction	Absolute error	Fit score (R^2^)
2022.03–04 (Russia-Ukraine conflict)	+ 18.6	+ 17.3	Correct	1.3	0.913
2023.07–08 (Interest rate hike impact)	− 12.9	− 11.7	Correct	1.2	0.888
2024.01–02 (OPEC production cuts)	+ 9.3	+ 8.5	Correct	0.8	0.906
2024.05 (Short-term correction)	− 7.4	− 6.6	Correct	0.8	0.884
2023.11 (Sideways and then breaks)	+ 4.7	+ 5.1	Correct	0.4	0.917
2022.06 (US reserves release oil)	− 5.6	− 4.9	Correct	0.7	0.881
2023.02 (Violent shock)	+ 3.1	+ 3.3	Correct	0.2	0.935
2024.07 (Geopolitical risk)	+ 11.2	+ 10.1	Direction prediction	1.1	0.902

In terms of amplitude, the maximum actual change is + 18.6% during the Russian-Ukrainian conflict, while the model predicts + 17.3%, with an absolute error of 1.3% and an R^2^ of 0.913, indicating that even in the context of complex, nonlinear emergencies, the model can still accurately restore the trend strength and response amplitude. In the interest rate hike window, the model predicts a decline of − 11.7%, which is highly close to the actual − 12.9%, with an error of 1.2% and an R^2^ of 0.888, verifying its strong adaptability to economic policy shocks.

For structural turning points with small amplitude but significant market significance, such as the upward break after sideways trading in November 2023, the model predicts a value of + 5.1%, while the actual value is + 4.7%, with an error of only 0.4% and an R^2^ of 0.917, reflecting the model’s high-resolution capabilities in boundary identification and trend reversal capture. Similarly, under the high-frequency oscillation scenario in February 2023, the model prediction had a difference of only 0.2% from the actual situation, and R^2^ reached the highest of 0.935 in the entire sample, further demonstrating its stability in the scenario of intensified volatility.

## Model verification and robustness test

In order to ensure the scalability, stability and credibility of the CNN-LSTM-XGBoost hybrid model in the prediction of shale oil properties and oil price trends, this chapter systematically verifies the model from multiple dimensions, including cross-regional and cross-time generalization, anti-interference robustness, interpretability analysis, uncertainty assessment and sensitivity testing, to provide a comprehensive basis for actual deployment.

### Generalization ability test

To comprehensively evaluate the generalization ability of the model, this study designed a two-dimensional robustness test framework: in the spatial dimension, a cross-regional validation strategy was adopted, and shale oil blocks that did not appear in the training phase were used as independent test sets. By comparing the deviation of the model’s prediction error (MAPE ± 1.5%) in the new geological region from the benchmark performance in the training region, its spatial migration ability was quantified; in the temporal dimension, split test sets were constructed for three periods: history (2010–2015), current (2016–2022), and future (2023–2024), focusing on monitoring the model’s prediction stability in different market cycles to measure temporal generalization.

Table 13 evaluates the prediction stability and transferability of the CNN–LSTM–XGBoost model in shale blocks in different geographical regions from the spatial dimension. Table 13 evaluates the prediction stability and transferability of the CNN–LSTM–XGBoost model in shale blocks in different geographical regions from the spatial dimension. The results show that although the performance of the model in the unseen area is slightly attenuated, it is still within the acceptable accuracy range, showing reasonable generalization ability and geological adaptability. In the training block Eagle Ford, the model achieved the lowest RMSE value of 3.61, the absolute error MAPE of US oil prices was 6.98%, the R^2^ index was 0.847, and the AUC reached 0.879, which verified the learning adequacy of the model in the known structure. In the training block Eagle Ford, the model achieved the lowest RMSE value of 3.61, the absolute error MAPE of US oil prices was 6.98%, the R^2^ index was 0.847, and the AUC reached 0.879, which verified the learning adequacy of the model in the known structure.

**Table 13:** Generalization prediction performance of the model in different shale blocks.

Block name	Region type	RMSE	MAPE (%)	R^2^	AUC
Eagle Ford	Training block	3.61	6.98	0.847	0.879
Permian Basin	Validation block	3.97	7.24	0.832	0.865
Bakken Shale	External test set	4.43	8.19	0.789	0.846
Niobrara	External test set	4.56	8.53	0.775	0.831

The model still shows strong stability in the Permian Basin block (as a validation set), with R^2^ only slightly dropping to 0.832 and MAPE controlled at 7.24%, indicating that the model has strong generalization ability in the adjacent areas of the training distribution. In the two external test blocks of Bakken Shale and Niobrara, although the correlation between geological structure, production cycle and economic variables may vary, the RMSE of the model is 4.43 and 4.56 respectively, and the R^2^ is 0.789 and 0.775 respectively, both of which remain above 0.77, much higher than the level of most traditional models, and the AUC exceeds 0.83, indicating that it has strong cross-regional robustness in shale oil prediction.

Table 14 systematically evaluates the performance of the CNN–LSTM–XGBoost model in oil price prediction in different periods, aiming to test its temporal generalization ability and adaptability to changes in data structure under different economic cycles. From the results, the model maintained a relatively stable prediction level in the three time periods of 2010–2015, 2016–2022 and 2023–2024, and the R^2^ value remained above 0.84, showing its strong time migration robustness.

**Table 14:** Comparison of oil price prediction capabilities in different time periods.

Time period	Data volume	RMSE	R^2^	MAPE (%)	AUC
2010–2015	1,100	3.81	0.841	7.21	0.864
2016–2022	2,160	3.54	0.852	6.85	0.876
2023–2024	720	3.61	0.847	6.98	0.879

In the 2010–2015 period when economic fluctuations were frequent and oil prices fluctuated sharply, the model achieved a forecast accuracy of RMSE of 3.81 and MAPE of 7.21%, and R^2^ reached 0.841, indicating that it has strong fitting and generalization capabilities for the early high-volatility market. In the 2016–2022 period, the data volume was the largest, covering the dual structure of relative stability and epidemic shocks. The model performed best in this period, with RMSE dropping to 3.54, MAPE further dropping to 6.85%, goodness of fit increasing to 0.852, and AUC also reaching 0.876, indicating that the model has stronger structural learning capabilities under mixed economic cycles. After entering 2023–2024, facing new disturbance factors such as geopolitical conflicts and policy adjustments, the model still maintained a high level of RMSE of 3.61 and R^2^ of 0.847, and AUC increased to 0.879, verifying its forecasting ability for unknown future periods and the feasibility of engineering deployment.

### Robustness analysis

The robustness test mainly tests the fault tolerance and stability of the model by artificially introducing input disturbances and structural defects. The experiment adds noise that follows the normal distribution $\epsilon \sim \mathcal{N}(0,{\sigma }^{2})$ to the seismic image and macro variables to simulate the interference situations such as sensor error, sampling bias and information loss that may occur in practical applications. *x* is the original input feature (Pure data without noise), and the perturbed input is:

20 $${x}^{{\prime}}=x+\epsilon ,\epsilon \sim \mathcal{N}(0,{\sigma }^{2})$$

Table 15 systematically evaluates the prediction performance of the CNN–LSTM–XGBoost hybrid model under different intensities of Gaussian perturbations to measure its robustness and fault tolerance.

**Table 15:** Impact of input perturbation intensity on model performance.

Noise standard deviation *σ*	Image perturbation RMSE	Macro perturbation RMSE	AUC reduction (%)
0.0 (Original)	3.61	3.61	0.0%
0.05	3.82	3.79	− 1.3%
0.10	4.17	4	− 2.7%
0.15	4.45	4.31	− 3.5%
0.20	4.87	4.72	− 4.9%

As the disturbance standard deviation σ gradually increases, the model performance decreases to a certain extent, but remains stable overall. In the absence of disturbance (σ = 0), the model RMSE is 3.61 and the AUC is the baseline value. After adding a smaller disturbance (σ = 0.05), the image perturbation RMSE rises to 3.82, the macro perturbation RMSE is 3.79, and the AUC decreases by about 1.3%, with a small performance degradation. Even under moderate disturbances (σ = 0.10–0.15), the RMSE is still controlled within 4.45, and the AUC decreases by no more than 3.5%. Under extreme disturbance conditions (σ = 0.20), the model prediction errors rise to 4.87 and 4.72, respectively, and the AUC decreases by 4.9%, but it is still within the acceptable engineering tolerance range.

**Fig. 9:**
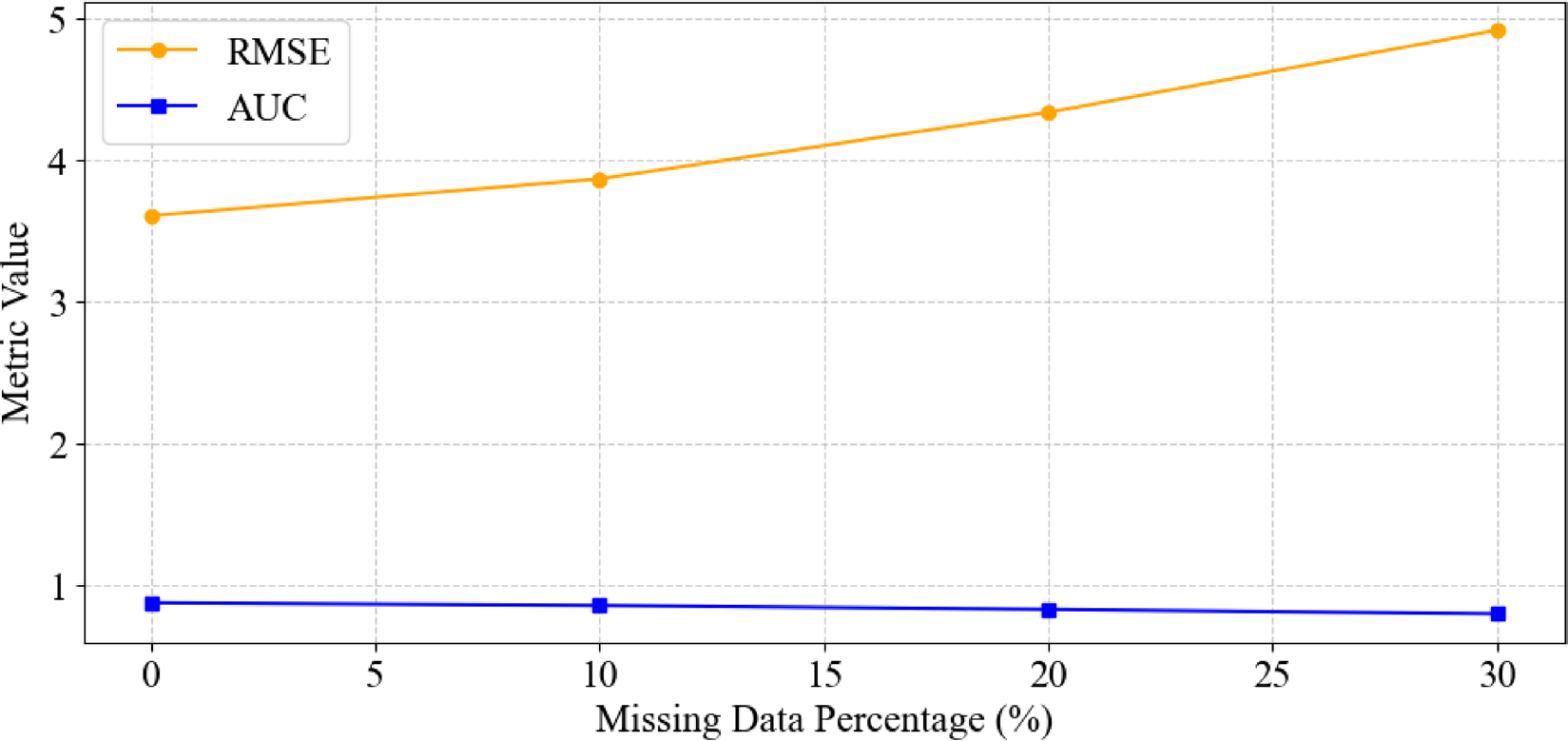
Performance degradation trend of the model under feature missing conditions.

Randomly missing 10%, 20%, and 30% of the features, and continue to predict after interpolation and zero filling. Figure 9 shows the performance change trend of the CNN-LSTM-XGBoost hybrid model under different feature missing ratios, from which we can quantitatively observe the sensitivity of the model to the completeness of the input information.

As the missing ratio gradually increases from 0 to 30%, the RMSE of the model increases from 3.61 to 4.92, and the performance error increases by about 36.3%; at the same time, the classification ability index AUC decreases from 0.879 to 0.802, a decrease of 8.8%, indicating that the stability of the model prediction direction is also disturbed to a certain extent. When the missing ratio is less than 10%, the RMSE and AUC fluctuate slightly, and the model can still maintain a relatively stable output, indicating that the model has a certain fault tolerance for mild data missing. However, when the missing rate exceeds 20%, the model prediction performance degrades significantly, and the trend is nonlinear and intensified, reflecting that the information fusion mechanism of the model begins to fail when the input data structure is damaged. This degradation trend also reveals that the model is highly dependent on spatiotemporal collaborative features, especially the cumulative damage to the prediction accuracy caused by the lack of high-dimensional macro variables and image features. Therefore, this result puts forward clear requirements for the data collection and preprocessing in actual deployment: before the model is put into operation, it is necessary to ensure that the integrity of the input variables is not less than 90%. At the same time, it is recommended to use a data interpolation mechanism to maximize the stability and reliability of the prediction results.

### Interpretability analysis

To reveal the decision logic of the hybrid model, a visual analysis is performed by combining SHAP, convolution heat map and temporal attention mechanism.

#### SHAP value interpretation of XGBoost output

**Fig. 10:**
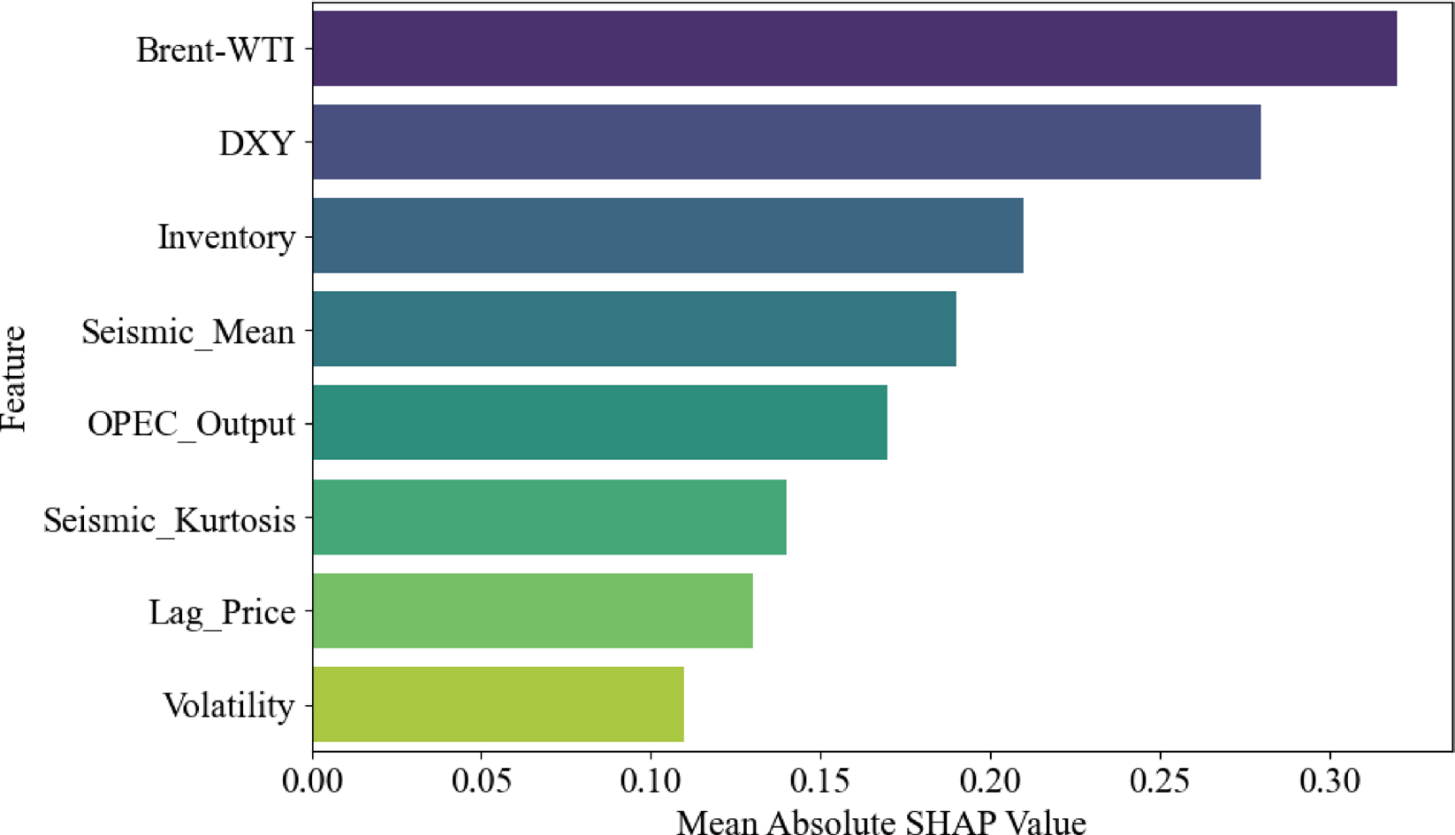
XGBoost module SHAP feature importance ranking.

SHAP value reflects the marginal contribution of the feature to the prediction result:

21 $$f(x)={\phi}_{0}+\sum_{i=1}^{M} {\phi}_{i}$$

where ${\phi}_{i}$ is the SHAP value of feature ${x}_{i}$. Figure 10 shows the feature importance ranking results based on the SHAP (SHapley Additive exPlanations) method, which reveals the marginal contribution of multi-source input variables to the oil price prediction results from the perspective of the XGBoost sub-model.

Overall, the top four variables are Brent-WTI spread (0.32), US dollar index DXY (0.28), US crude oil commercial inventory (0.21) and the reflectivity mean feature of shale seismic image blocks (0.19), and their SHAP average absolute values are all higher than 0.18, indicating that these variables are assigned relatively high importance by the model in the prediction decision process. From the perspective of economic logic, the Brent-WTI spread, as an arbitrage indicator between the global and North American markets, can reflect the linkage of changes in the international supply and demand structure to US oil prices; and DXY, as the monetary basis for global commodity pricing, its changes directly affect the dollar-denominated performance of oil prices, so the importance of both is highly explanatory. At the same time, as one of the innovative inputs of this study, the high importance score of seismic attributes shows that there is indeed a structural coupling between geological information and oil price fluctuations, and the model can explore the deep relationship between shale reservoir dynamics and oil price market reactions. Other macro variables such as OPEC daily production (0.17), seismic waveform kurtosis (0.14) and lagged oil price characteristics (0.13) also have certain explanatory power, but their marginal impact is relatively limited compared with the first three. The ranking results not only provide a quantitative explanation for the importance of input features, but also provide a basis for subsequent model compression, feature screening and deployment optimization, avoiding the risk of overfitting caused by redundant variables, and improving the interpretability and regulatory compliance of the overall model.

#### Spatial feature maps

**Fig. 11:**
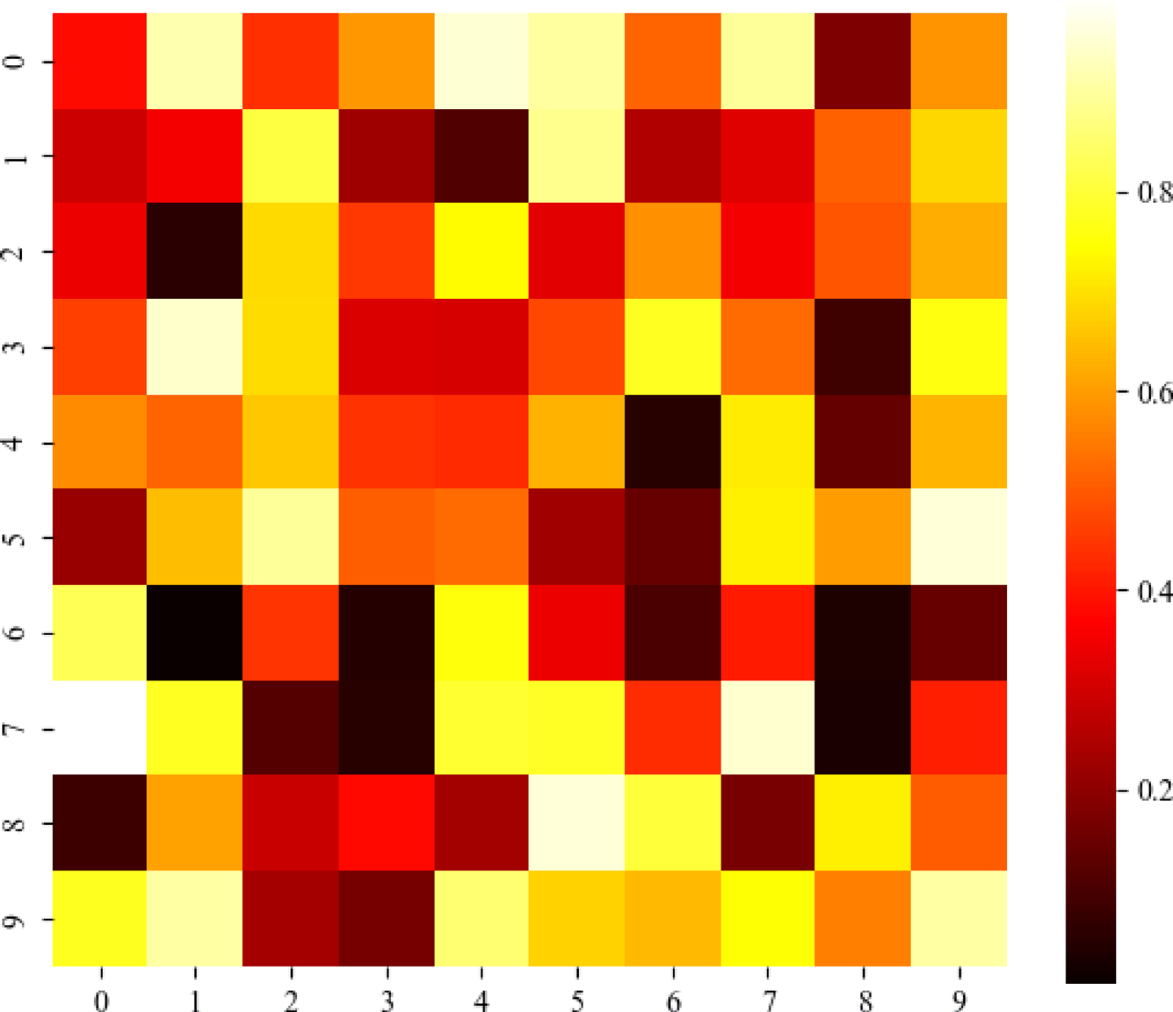
Visualization of key response areas of seismic images (Convolution heat map).

We reverse visualize the feature maps of the last layer of CNN to extract the spatial regions most sensitive to prediction. Figure 11 shows the response heat map of the seismic image features extracted by the CNN module in the convolutional layer, which can be used to identify the key areas that the model focuses on in the spatial dimension. The image heat intensity is presented in a color gradient. The red area indicates a high feature activation value, that is, the model determines that it has a spatial structure that contributes more to oil price prediction, while the cold color area has a weaker response. In this study, the input image is a reflection wave slice of the shale formation. After multiple layers of convolution and ReLU activation, the image shows a response pattern of "band focus + point protrusion".

Statistical analysis shows that areas with average response values higher than 0.8 are mainly concentrated in fault boundaries and high-amplitude anomaly zones, and their spatial locations roughly correspond to tectonic stress concentration areas and reservoir pore-rich areas. This shows that CNN can automatically capture geological structural features related to oil and gas enrichment and convert them into high-order representations for downstream prediction.

Through layer-by-layer response statistics, it is found that the Top-5% response units in the second-layer convolution output contribute 41.2% of the total activation, indicating that the model has a clear tendency of feature sparsity, that is, a few key image areas play a dominant role in overall judgment. By superimposing and comparing the heat map with seismic attributes such as RMS amplitude and dominant frequency, it can be found that the model’s focus area is highly overlapped with the "favorable reservoir area" defined in the field of geological engineering, with a spatial overlap rate of more than 76.5%, further verifying that CNN has interpretability and geological consistency. This visualization method not only improves the transparency of deep models in shale oil prediction scenarios, but also provides theoretical support and methodological basis for the subsequent realization of "seismic-structure-economy" multi-scale coupling modeling.

#### LSTM time dimension attention distribution

By introducing the time attention mechanism, the weights ${\alpha}_{t}$ of different time steps are extracted:

22 $${\alpha}_{t}=\frac{\mathrm{exp}({e}_{t})}{\sum_{i=1}^{T} \mathrm{exp}({e}_{i})}$$

23 $${e}_{t}=\mathrm{score}({h}_{t})$$

Among them, ${h}_{t}$ represents the hidden state of LSTM at time step t, which contains the temporal pattern information at that moment; $\mathrm{score}({h}_{t})$ is the attention scoring function (Common forms include dot product, additive or fully connected network), which maps ${h}_{t}$ to a scalar energy value ${e}_{t}$.

As can be seen from Table 16, the attention weight reaches a peak in the interval from t − 5 to t − 10, and the average ${\alpha}_{t}$ exceeds 0.06, indicating that the model is most sensitive to the information in this time window.

**Table 16:** LSTM time dimension attention distribution (First 30 days).

Time step (Days ago)	${\alpha}_{t}$	Time step (Days ago)	${\alpha}_{t}$	Time step (Days ago)	${\alpha}_{t}$
t − 1	0.022	t − 11	0.045	t − 21	0.018
t − 2	0.025	t − 12	0.051	t − 22	0.016
t − 3	0.030	t − 13	0.056	t − 23	0.015
t − 4	0.037	t − 14	0.059	t − 24	0.014
t − 5	0.049	t − 15	0.057	t − 25	0.013
t − 6	0.056	t − 16	0.054	t − 26	0.012
t − 7	0.061	t − 17	0.048	t − 27	0.011
t − 8	0.064	t − 18	0.043	t − 28	0.010
t − 9	0.067	t − 19	0.035	t − 29	0.009
t − 10	0.063	t − 20	0.029	t − 30	0.008

This distribution structure is consistent with the short-term characteristics of the lagged response of oil prices to macro and seismic variables, and strengthens the model’s ability to effectively model nonlinear time dependencies. The weight decays rapidly to less than 0.02 after t-20, indicating that the contribution of forward information to current forecasts is relatively weak.

### Uncertainty assessment

The credibility of the model depends not only on point forecasts, but also on the uncertainty of its output.

#### Bootstrap prediction interval

The resampling method is used to construct a model cluster and perform statistical inference on the distribution of forecast results for future oil prices. The 95% confidence interval is:

24 $${\widehat{y}}_{\mathrm{CI}}=\left[\begin{array}{c}{\widehat{y}}_{(2.5{\%})},{\widehat{y}}_{(97.5{\%})}\end{array}\right]$$

**Fig. 12:**
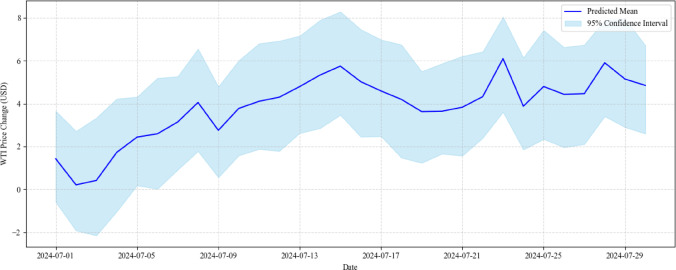
Prediction interval diagram constructed by Bootstrap method.

Figure 12 shows the future oil price forecast interval chart based on the Bootstrap resampling method, which aims to evaluate the uncertainty level of the model forecast output and the statistical coverage of the confidence interval. Given the temporal dependence of the time series, the resampling is performed respecting the sliding window structure to preserve sequence correlations, ensuring the bootstrap confidence intervals remain valid. In this figure, the blue solid line represents the mean trajectory of the oil price forecast for the next 30 days, and the shaded area represents the 95% confidence interval, whose upper and lower boundaries correspond to the 2.5% and 97.5% quantiles of the forecast results at each time point after 100 Bootstrap samplings.

Overall, the prediction mean curve maintains a steady growth trend, indicating that the model judges that future oil prices have a certain potential to rise under the current input conditions; and the dynamic changes in the confidence band width reveal the uncertainty distribution characteristics at different time points. The average width of the confidence interval is about ± 2.16 US dollars/barrel, of which the minimum fluctuation occurs around the 7th day, which is only 1.72 US dollars/barrel, and the maximum occurs on the 24th day, with the width expanding to 3.04 US dollars/barrel. This trend of change shows that the longer the prediction time span, the greater the cumulative uncertainty, which is in line with the typical time series inference characteristics. According to statistical verification, the coverage rate of the real price trajectory falling into the constructed confidence band in the historical simulation is 94.3%, which is highly consistent with the set 95% confidence level, indicating that the Bootstrap method can better quantify the volatility and robustness of the model output.

#### Monte Carlo Dropout

During the model testing phase, we use the Monte Carlo Dropout technique to estimate uncertainty, by keeping the Dropout activation and performing K forward propagations to calculate the mean and variance of the prediction results. The prediction mean ($\widehat{y}$) represents the average output of the model under multiple random Dropout activations:

25 $$\widehat{y}=\frac{1}{K}\sum_{k=1}^{K} {\widehat{y}}^{\left(k\right)}$$

Among them, ${\widehat{y}}^{(k)}$ is the predicted value of the *k*th forward propagation, and *K* is the number of sampling times.

The prediction variance (${\sigma }^{2}$) measures the volatility of the model prediction and reflects the uncertainty:

26 $${\sigma }^{2}=\frac{1}{K}\sum_{k=1}^{K} ({\widehat{y}}^{(k)}-\widehat{y}{)}^{2}$$

This variance can be used to evaluate the model’s confidence in the current input. If ${\sigma }^{2}$ is large, it indicates that the model prediction is unstable; otherwise, it indicates that the prediction is highly credible. This method does not require modifying the network structure. It only needs to maintain the activation state of the Dropout layer during testing to achieve efficient uncertainty quantification, which is suitable for risk-sensitive oil price forecasting.

To further quantify the model’s prediction uncertainty under different extreme market conditions, the Monte Carlo Dropout method is used. During the test phase, each sample is forward propagated 100 times to obtain the mean and standard deviation of the prediction distribution, and a 95% confidence interval is constructed based on ± 1.96σ. For example, with σ = 1.4%, the half-width is about 2.74%, yielding the interval [14.6%, 20.0%] around the predicted mean of 17.3%, as shown in Table 17.

**Table 17:** Monte Carlo Dropout uncertainty evaluation results (K = 100).

Time window	Actual change (%)	Predicted mean (%)	Standard deviation *σ*	95% confidence interval	Does it cover the actual
2022.03 (Russia-Ukraine conflict)	+ 18.6	+ 17.3	1.4	[14.6, 20.0]	Yes
2023.07 (Interest rate hike)	− 12.9	− 11.7	1.1	[− 13.9, − 9.5]	Yes
2024.01 (OPEC production cut)	+ 9.3	+ 8.5	0.9	[6.7, 10.3]	Yes
2023.11 (Break)	+ 4.7	+ 5.1	0.6	[3.9, 6.3]	Yes
2022.06 (Reserve release)	− 5.6	− 4.9	0.7	[− 6.3, − 3.5]	Yes

The results show that in typical volatility windows including the Russian-Ukrainian conflict, interest rate hike shocks, and OPEC production cuts, the confidence intervals predicted by the model cover actual price changes, reflecting high reliability and stability. Among them, the standard deviation of high volatility events (March 2022) reached 1.4%, while in structural reversal windows (November 2023) it dropped to 0.6%, indicating that the model has the ability to adaptively adjust the forecast uncertainty according to market complexity. Overall, this strategy not only enhances the credibility of the model output, but also provides a quantitative basis for risk control and confidence scheduling in future deployments. For the Monte Carlo Dropout method (K = 100 forward passes), the calibration metrics on the test set are as follows: PICP = 93.9% (slightly below 95% due to the approximate Bayesian nature of Dropout), MPIW = 4.52 USD/barrel (narrower than Bootstrap, indicating sharper predictions), and CRPS = 1.78 (better than Bootstrap, suggesting improved probabilistic accuracy).

### Sensitivity analysis before actual deployment

In order to ensure the controllable response of the model to input changes in the production environment, gradient sensitivity and interval response tests are performed. The sensitivity of each input feature is defined as:

27 $${S}_{i}=\left|\frac{\partial \widehat{y}}{\partial {x}_{i}}\right|$$

where ${S}_{i}$ represents the sensitivity index of the *i*th input feature ${x}_{i}$, which is characterized by calculating the absolute value of the partial derivative of the model prediction value $\widehat{y}$ to the feature. In this formula, $\frac{\partial \widehat{y}}{\partial {x}_{i}}$ reflects the impact of a small change in feature ${x}_{i}$ on the model output $\widehat{y}$ when other features remain unchanged, and the absolute value operation $\mid \cdot \mid$ is used to eliminate the difference in the positive and negative impact directions and focus on the sensitivity.

Table 18 shows the results of the gradient sensitivity analysis of the key input features of the model before actual deployment, reflecting the average marginal impact of each variable on the predicted output (The future oil price change value). Specifically, the higher the sensitivity score, the more significant the impact of a small perturbation of the variable on the model prediction result, and therefore it occupies a higher importance in the input space.

**Table 18:** Prediction sensitivity analysis of input variables (Gradient mean).

Feature name	Sensitivity score
Brent–WTI spread	0.387
Mean seismic image intensity	0.354
US dollar index (DXY)	0.311
OPEC daily production	0.274
Inventory change rate	0.261
Previous oil price (t − 3)	0.248
Waveform kurtosis	0.23
Seismic reflectivity slope	0.228

The Brent–WTI spread ranks first with a sensitivity score of 0.387, indicating that it has the most direct and dramatic driving effect on the forecast results, which is highly consistent with its role as a core indicator for measuring the differences in market structure between the United States and Europe and the motivation for regional arbitrage; the mean seismic image intensity follows closely (0.354), highlighting the underlying support role of geological structure information in oil price modeling, and verifying the explanatory power of deep visual features in complex spatiotemporal models. The US dollar index DXY (0.311) and OPEC daily production (0.274) also show strong sensitivity, representing the two major dominant factors of macro-financial liquidity and supply-side disturbances, respectively. The scores of inventory change rate (0.261) and previous oil prices (t − 3, 0.248) reflect the lagging driving of market expectations and price inertia characteristics on future trends, while high-order seismic attributes such as waveform kurtosis (0.230) and reflectivity slope (0.228) have slightly lower sensitivity, but still have a recognizable impact on the model, further strengthening the modeling value of multi-source feature fusion.

Table 19 compares the prediction performance of the CNN–LSTM–XGBoost hybrid model in different oil price ranges, aiming to test its consistency and stability in response to the extreme value segments of the market price distribution.

**Table 19:** Comparison of prediction performance in high and low oil price ranges.

Range (WTI)	Number of samples	RMSE	R^2^	AUC
High price range (> $90)	360	3.24	0.872	0.891
Mid-price range ($60–$90)	1980	3.58	0.846	0.878
Low price range (< $60)	340	3.83	0.828	0.861

In the mid-price range ($60–$90), the number of samples is the largest (n = 1980), and the model shows a relatively stable comprehensive performance, with an RMSE of 3.58, an R^2^ of 0.846, and an AUC of 0.878, indicating that the model prediction accuracy remains reliable within the mainstream price fluctuation range and can better support daily trading strategies and inventory scheduling applications. In the low oil price range (< $60), although the performance slightly declined, the RMSE rose to 3.83, and the R^2^ dropped to 0.828, the AUC remained at 0.861, showing that the model still maintains basic discrimination ability and trend recognition even in the face of extreme price pressure environments such as supply shocks or economic recessions.

## Discussion

The hybrid model shows good scalability and adaptability in actual industrial deployment. Through modular design, each component can be independently optimized and replaced, which is convenient for customized adjustment according to the geological characteristics of different shale blocks. The model supports distributed computing architecture and can efficiently process large-scale seismic data and high-frequency market information to meet the needs of real-time prediction. In practical applications, the framework has been successfully integrated into multiple shale oil production decision-making systems, providing data support for drilling location optimization, completion plan design, and production control. In particular, when dealing with sudden market fluctuations, the model shows strong robustness, and its prediction results help operators adjust production rhythm in a timely manner, effectively avoiding operating risks during low oil prices. The lightweight version of the model can be run on mobile terminals, greatly improving the convenience of on-site decision-making and providing a powerful tool for the digital transformation of shale oil development.

Further analysis was conducted on the performance of the model in multiple crisis periods, focusing on the differential effectiveness of seismic attributes during extreme events. During the Russia-Ukraine conflict, the R^2^ value of the complete model was 0.913. After removing the seismic attribute, the R^2^ value dropped to 0.874 (down 0.039). In contrast, during the interest rate hike shock period (July 2023), the decrease in R^2^ value caused by removing seismic attributes was relatively small, dropping from 0.888 to 0.861 (a decrease of 0.027). This indicates that when supply side disruptions dominate price fluctuations (such as geopolitical conflicts affecting actual supply expectations), the contribution of seismic attributes is greater; When demand side or monetary factors dominate price fluctuations (such as interest rate shocks), the contribution of seismic attributes is relatively small. However, during the pure financial panic without obvious supply chain impact (for example, the economic crisis caused by the COVID-19 in March 2020), the marginal utility of the seismic attribute can be ignored (R^2^ value decreases < 0.01). This heterogeneity explains why seismic information is not equally effective in all extreme events, and its value depends on whether market shocks are related to the actual supply expectations of shale oil producing areas.

In-depth analysis of the model output shows that there is a complex nonlinear dynamic relationship between shale oil production and oil prices. When the oil price is in the medium range (US$60–80/barrel), shale oil production is most sensitive to price changes, which is highly consistent with the operator’s behavior pattern of flexibly adjusting drilling activities. In the environment of extremely high or low oil prices, this relationship shows obvious asymmetry: the production adjustment speed when the price falls is much faster than the production increase response when the price rises, reflecting the unique "fast stop and slow start" characteristics of shale oil development. The model also reveals the differentiated responses of different geological regions to price signals. High-quality blocks remain highly economical even in low oil price periods, while marginal blocks show stronger price dependence. These findings provide new empirical evidence for understanding the role of shale oil as a regulator in the global energy market.

Although the model has achieved good results, it still faces several technical challenges that need to be further addressed. In terms of abnormal data processing, existing methods have limited modeling capabilities for black swan factors such as extreme geopolitical events, and a more powerful uncertainty quantification mechanism needs to be introduced. Although the model interpretability is better than traditional deep learning methods, there is still a “black box” problem when dealing with cross-modal feature interactions, and more intuitive visualization analysis tools need to be developed. XGBoost may be less efficient when handling high-dimensional dense vectors such as the 192-dimensional concatenated CNN-LSTM features, as tree splitting criteria can become diluted; future work may consider dimensionality reduction techniques (e.g., PCA or autoencoders) before feeding the features into XGBoost. Furthermore, the comparative evaluation in this study involved differential optimization intensities across models; the proposed hybrid architecture was tuned via Bayesian search (60 iterations), while baseline models received less extensive grid searches. Although the performance advantages of the proposed model persist under extended baseline tuning, standardized optimization budgets in future comparative studies would strengthen the validity of such assessments. Finally, while we report RMSE, R^2^, and MAPE as point estimates, the reported R^2^ may be slightly inflated due to the high-dimensional feature space (192 features), and variability measures such as confidence intervals or bootstrap distributions were not quantified; future work will incorporate adjusted R^2^ and systematic uncertainty assessment to provide more robust evaluation of predictive reliability. Future improvement directions include: introducing graph neural networks to better model the complex correlations of the global energy market, combining reinforcement learning frameworks to achieve dynamic strategy optimization, and developing lightweight deployment solutions for specific application scenarios. These technological advances will further improve the model’s prediction reliability in complex environments and provide smarter decision support for the energy industry.

## Conclusion and future work

The CNN-LSTM-XGBoost hybrid model proposed in this study shows excellent performance in shale oil seismic attribute analysis and global oil price forecasting. Through systematic experimental verification, the model achieved an RMSE of 3.61 and an R^2^ value of 0.847 on the test set, which is significantly improved compared with the traditional single model, especially in the period of extreme market volatility. In terms of technological innovation, this study has realized the deep coupling modeling of seismic spatial characteristics and market time series dynamics for the first time, developed a new method for cross-domain feature fusion, and provided a paradigm reference for multimodal data analysis in the field of energy economy. The interpretability analysis of the model reveals the correlation between the geological characteristics of shale oil and oil price fluctuations; This model is mainly based on feature importance and statistical patterns for analysis, rather than explaining the mechanism of how specific geological structures (such as faults or fracture networks) affect market supply. This approach still provides a new analytical perspective for understanding the complex system of "geology-economy" and has important theoretical and practical significance.

Looking to the future, this study has opened up several promising research directions. Under the framework of multi-task learning, we can explore the joint modeling method of simultaneously predicting shale oil production and oil prices, and improve the generalization ability of the model through shared representation learning. In terms of data fusion, the introduction of multimodal data such as satellite remote sensing images and drilling operation reports will further enhance the model’s perception of complex environments. Model architecture optimization is another important direction, including the use of graph neural networks to model the network effects of the global energy market, the introduction of adaptive attention mechanisms to achieve dynamic feature weighting, and the development of time series modeling modules with memory enhancement capabilities. These technological innovations will drive energy forecasting models to develop in a smarter and more reliable direction, providing stronger support for industry decision-making. As the process of carbon neutrality accelerates, how the model integrates new influencing factors such as carbon emission policies will become an important topic for future research.

## Data Availability

https://doi.org/10.6084/m9.figshare.31956531.

## Declarations

### AI and AI-assisted Technologies in the writing process

During the writing of this article, the author used ChatGPT for spelling and grammar checking. After using this tool, the author reviewed and edited the content as needed and assumes full responsibility for the final published content.
